# Tailoring the photoelectrochemistry of catalytic metal-insulator-semiconductor (MIS) photoanodes by a dissolution method

**DOI:** 10.1038/s41467-019-11432-1

**Published:** 2019-08-06

**Authors:** G. Loget, C. Mériadec, V. Dorcet, B. Fabre, A. Vacher, S. Fryars, S. Ababou-Girard

**Affiliations:** 1 Univ Rennes, CNRS, ISCR (Institut des Sciences Chimiques de Rennes)-UMR6226-ScanMAT-UMS2001, F-35000 Rennes, France; 2 Univ Rennes, CNRS, IPR (Institut de Physique de Rennes)-UMR 6251, F-35000 Rennes, France

**Keywords:** Surface chemistry, Electrocatalysis, Solar fuels, Photocatalysis

## Abstract

Apart from being key structures of modern microelectronics, metal-insulator-semiconductor (MIS) junctions are highly promising electrodes for artificial leaves, i.e. photoelectrochemical cells that can convert sunlight into energy-rich fuels. Here, we demonstrate that homogeneous Si/SiO_x_/Ni MIS junctions, employed as photoanodes, can be functionalized with a redox-active species and simultaneously converted into high-photovoltage inhomogeneous MIS junctions by electrochemical dissolution. We also report on the considerable enhancement of performance towards urea oxidation, induced by this process. Finally, we demonstrate that both phenomena can be employed synergistically to design highly-efficient Si-based photoanodes. These findings open doors for the manufacturing of artificial leaves that can generate H_2_ under solar illumination using contaminated water.

## Abstract (web summary)

Designing synthetic systems to convert light into fuel is crucial in renewable energy development. Here, authors study electrodissolution in nickel thin films from metal-insulator-semiconductor junctions and find decreased homogeneity to improve junction properties and catalytic performances.

## Introduction

Sunlight can be directly converted into H_2_ by photoelectrochemical cells (PECs, *a.k.a*. artificial leaves), devices that are based on semiconductor (SC) photoelectrodes, immersed in water, that mimic photosynthesis by converting sun’s photons into energy-rich molecules^1–3^. The operating principle of PECs relies on the photogeneration of electron-hole pairs (*e*^*−*^-*h*^*+*^) in the SC under solar illumination, the separation of these charge carriers and their transportation at the solid/liquid interface where they react with water. Photogenerated *e*^*−*^ are employed for the hydrogen evolution reaction (HER, reaction (1)), producing highly pure H_2_ that can be stored and employed on demand to generate clean electricity. In order to respect electroneutrality, the photogenerated *h*^*+*^ must be consumed for a counter-reaction, which is generally the oxygen evolution reaction (OER, reaction (2))^4,5^. Unfortunately, the OER requires a considerable energy input (*E*^0^_O2/H2O_ = 1.23 V vs reversible hydrogen electrode RHE), which limits the overall efficiency of the PEC and generates a low-value product (O_2_). Based on these considerations, it is straightforward that replacing the OER by an electrochemical reaction more valuable or/and proceeding at a lower energy cost would be highly beneficial to increase the efficiency and the added value of PECs^6^. A very appealing reaction that can be employed at the photoanode side is the urea oxidation reaction (UOR, reaction (3)), which can be triggered at a lower potential than OER on appropriate electrocatalysts^7–9^. Furthermore, very little has been reported on photoelectrochemical UOR^10^, which is surprising because this reaction allows degrading urea, the main constituent of urine, one of the most abundant wastes on Earth^7,11^.

1 $$\mathrm{HER}:2\;\mathrm{H}_{2}\mathrm{O}\left( \mathrm{l} \right) + 2\;\mathrm{e}^ {-} \to \mathrm{H}_{2}\left( \mathrm{g} \right) + 2\;\mathrm{OH}^ {-} \left( \mathrm{aq} \right)$$

2 $$\mathrm{OER}:2\;\mathrm{OH}^ {-} \left( \mathrm{aq} \right) + 2\;\mathrm{h}^ {+} \to 1/2\;\mathrm{O}_{2}\left( \mathrm{g} \right) + \mathrm{H}_{2}\mathrm{O}\left( \mathrm{l} \right)$$

3 $${{\mathrm{UOR}:\left( \mathrm{NH}_{2} \right)_{2}\mathrm{CO}\left( \mathrm{aq} \right) + 6\;\mathrm{OH}^ {-} \left( \mathrm{aq} \right) + 6\;\mathrm{h}^ {+} \to \mathrm{N}_{2}\left( \mathrm{g} \right) + 5\;\mathrm{H}_{2}\mathrm{O}\left( \mathrm{l} \right) + \mathrm{CO}_{2}\left( \mathrm{g} \right)}}$$

Besides, careful consideration of material aspects is crucial for PEC design. In particular, photoelectrodes must: absorb a large portion of the solar spectrum (i.e. have a short band gap (*E*_g_)), have a high charge carrier lifetime and be inexpensive. In addition to fulfilling these criteria, Si (*E*_g_ = 1.1 eV) is highly abundant (the 2nd element on the earth crust), nontoxic and widely employed by the microelectronics and photovoltaic industries, which makes it a prime candidate to be employed for PEC manufacturing^12^. On the other hand, using Si as a photoelectrode material is a real challenge because of its poor catalytic activity and its notorious instability in aqueous solution^13^. Two main deleterious mechanisms are known to occur on Si-based photoelectrodes, the first one, very pronounced at the photoanode, is the self-oxidation of Si into SiO_x_, which electrically insulates the SC and inhibits charge transfer at the solid/electrolyte interface. The second deactivation process is the chemical etching of Si which is known to occur spontaneously at high pH. Recent remarkable research works revealed that the efficiency and the stability of Si-based OER photoanodes can be considerably increased by incorporating protective and catalytic coatings, such as Ni thin films^14,15^. Interestingly, several studies have reported that the operation of Ni-coated Si photoanodes at high pH could lead to considerable performance improvement^14,16^. This process, usually referred as activation, modifies simultaneously the structure as well as the chemical composition of the interface, making difficult to precisely differentiate the effect of single parameters on photovoltage and catalysis. For instance, several hypotheses can be made to explain the enhancement in photovoltage, including oxidation of the Ni layer^17^, partial equilibration of the *n*-Si with the liquid phase^14^ and increase of inhomogeneity in the Ni thin film^16^. In this frame, Si photoanodes based on the metal-insulator-SC (MIS) junction, a key structure of modern electronics, are attracting considerable attention, owing to their remarkable photoelectrochemical properties^18,19^. Of particular interest, it has been reported lately that inhomogeneous MIS photoanodes^20,21^, manufactured by bottom-up wet processes (e.g. electrodeposition) on *n*-type Si (*n*-Si) surfaces can be used to efficiently oxidize water at high pH^22^.

In this article, we report a fundamentally different top-down approach, based on scalable electrodissolution for converting poorly-active homogeneous Si/SiO_x_/Ni to highly active inhomogeneous Si/SiO_x_/Ni MIS photoanodes. In addition to allowing precise control over the junction inhomogeneity, this method modifies the surface with a redox-active Ni-Prussian blue derivative (NiFePB) which allows a direct probing of the junction properties. We show that the increased inhomogeneity improves the photovoltage (*V*_oc_) and we demonstrate that the so-processed layers exhibit a pronounced electrocatalytic activity for UOR, which is rationally elucidated. We finally show how electrodissolution can generate synergistic effects in terms of photovoltage and catalytic activity, which allows, in fine, designing efficient UOR photoanodes only composed of highly abundant materials. These results bring new opportunities for the development of PECs that can produce H_2_ using urea-contaminated water and solar illumination.

## Results

### Preparation and characterization of the surfaces

**Fig. 1:**
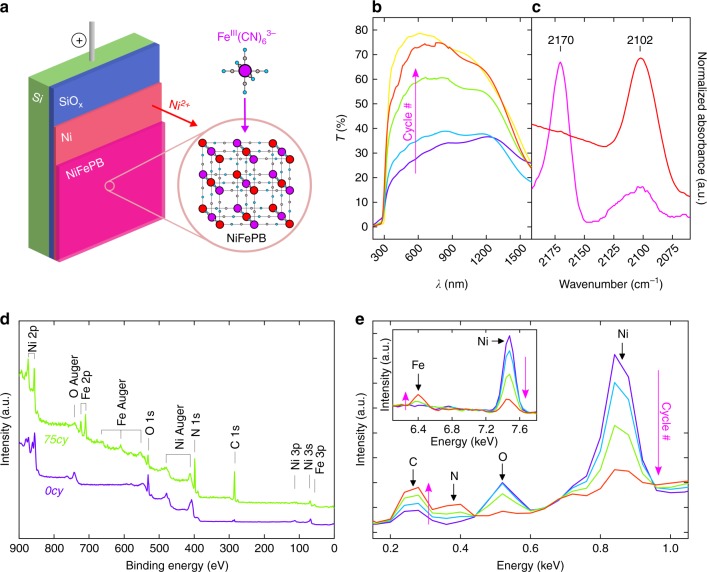
Physicochemical characterization of the Ni/NiFePB layers. **a** Scheme showing the method employed for manufacturing the Si/SiO_x_/Ni/NiFePB surfaces. **b** Transmittance spectra of FTO/Ni/NiFePB surfaces obtained with different number of cycles ((purple) *0cy*, (light blue) *25cy*, (light green) *75cy*, (red) *150cy*, (yellow) bare FTO). **c** ATR-FTIR spectra of a *p*^*+*^-Si/SiO_x_/Ni/NiFePB surface (pink) after preparation and (red) after cathodic polarization in 1 M KCl. **d** XPS survey spectra recorded for (purple) *p*^*+*^-Si/SiO_x_/Ni/NiFePB-*0cy* and (light green) *p*^*+*^-Si/SiO_x_/Ni/NiFePB-*75cy*. **e** EDS spectra of *p*^*+*^-Si/SiO_x_/Ni/NiFePB obtained with different number of cycles (purple) *0cy*, (light blue) *25cy*, (light green) *75cy*, and (red) *150cy*)

The homogeneous Si/SiO_x_/Ni MIS electrodes used as substrates were prepared, first, by chemically oxidizing Si (100) wafers to create a 1.5-nm-thick SiO_x_ tunnel layer (as determined by ellipsometry measurements, see Supplementary Fig. 1). After that, a 25-nm-thick Ni layer was deposited on SiO_x_ by DC magnetron sputtering. The so-prepared Si/SiO_x_/Ni MIS electrodes were then modified with NiFePB by electrochemically oxidizing the Ni layer in the presence of Fe(CN)_6_^3−^ at pH 2.5 (Fig. 1a). Indeed, when an anodic current is imposed, Ni^0^ is converted in soluble Ni^2+^, which coordinates with the cyano groups of Fe(CN)_6_^3−^ to generate the NiFePB coating at the solid interface. We employed sequential current cycles comprising an anodic pulse (1 s) followed by a resting time (3 s) to replenish the diffusion layer with Fe(CN)_6_^3−^ (Supplementary Fig. 2 shows the chronopotentiometry curves). In the following, we refer to the NiFePB coatings as NiFePB-*#cy*, with *#cy* being the number of imposed electrochemical cycles. This procedure had a pronounced optical effect on the Ni layer, which was studied by employing transparent fluorine-doped tin oxide (FTO)-coated glass slides as substrates, processed in the same manner as for Si/SiO_x_ surfaces. The transmittance of electrodes modified with 25, 75, and 150 electrochemical cycles is reported in Fig. 1b. These spectra and the corresponding optical photographs (Supplementary Fig. 3) show that the layer transmittance increased with the number of electrochemical cycles, well in line with the consumption of the Ni layer. The transmittance maxima varied from 37% for a non-processed FTO/Ni surface to 75% for FTO/Ni/NiFePB-*150cy* (close to the 78% obtained for bare FTO), which demonstrates that the method improves light transmission through the Ni film, an important parameter for photoelectrode design.

The presence of CN bonds on the surface after modification was evidenced by attenuated total reflectance Fourier transform infrared spectroscopy (ATR-FTIR), recorded on *p*^*+*^-Si/SiO_x_/Ni/NiFePB in dry air (Fig. 1c). After modification (pink curve), the main band in the 2200–2050 cm^-1^ region was observed at 2170 cm^−1^, in close agreement with the reported values for CN bridging ligands in Prussian blue^23^, KNi^II^Fe^III^(CN)_6_ and NiFePB^24^. A smaller band was also observed at 2105 cm^−1^, which is close to the value reported for K_2_Ni^II^Fe^II^(CN)_6_^24^ and likely indicates the presence of a small amount of Fe^II^ in the coating after preparation. Remarkably, after cathodic polarization at +0.2 V vs KCl-saturated calomel electrode (SCE) for 30 s (Fig. 1c, red curve), only one band was observed at 2102 cm^−1^, confirming the full electrochemical conversion of Fe^III^ to Fe^II^ in the NiFePB coating. The composition of the outermost electrode surface was analyzed by X-ray photoelectron spectroscopy (XPS, Fig. 1d) before modification (*p*^*+*^-Si/SiO_x_/Ni/NiFePB-*0cy*, violet curve) and after modification (*p*^*+*^-Si/SiO_x_/Ni/NiFePB-*75cy*, green curve). The survey spectra confirmed the presence of all expected elements, i.e., Ni and O for the unmodified surface and Ni, O, Fe, N, and C for the modified surface. The presence of oxygen is attributed to the native NiO/Ni(OH)_2_ layer that spontaneously forms over Ni at ambient conditions^25^, which was confirmed by further XPS experiments, described in the SI (Supplementary Fig. 4 and Supplementary Note 1). After modification, the Ni 2p region (Supplementary Fig. 5) exhibited a main peak at 856.3 eV, in good agreement with the Ni 2p_3/2_ value expected for a NiFePB layer^26^. In addition, analysis of the Fe 2p region (Supplementary Fig. 6) confirmed the presence of a major Fe^III^ component and a minor Fe^II^ component in the NiFePB after preparation, with 2p_3/2_ binding energies respectively located at 709.8 and 708.4 eV. Furthermore, energy-dispersive X-ray spectroscopy (EDS) allowed investigating the bulk composition of the thin film as a function of the number of electrochemical modification cycles (Fig. 1e, Supplementary Figs. 7 and 8). As shown in this data, the C, N, and Fe contents increased with the number of cycles; confirming the modification of the Ni surface with the NiFePB layer.

**Fig. 2:**
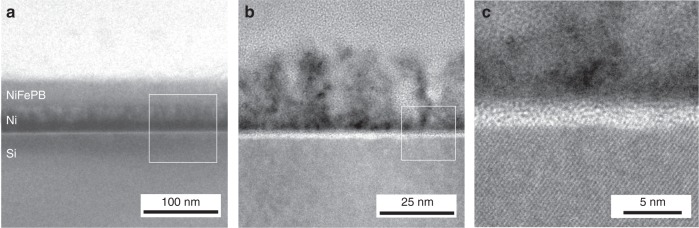
Characterization of the Si/SiO_x_/Ni/NiFePB interface. Cross-sectional TEM images of *p*^*+*^-Si/SiO_x_/Ni/NiFePB-*75cy*. The white square in **a** indicates the interface that is imaged in **b** and the white square in **b** indicates the interface that is imaged in **c**

The morphological characterization was performed by electron microscopy. A careful analysis of the scanning electron microscopy (SEM) top-view images (Supplementary Figs. 9–12) obtained for *0cy*, *25cy*, *75cy*, and *150cy* allowed elucidating the structure of the coating, which was composed of a NiFePB layer covering the Ni thin film. The NiFePB layer was clearly observed but was, however, ruptured by cracks, likely caused by the surface drying, which allowed observing the morphology of the underlying Ni thin film. Raising the number of cycles increased the porosity of the Ni film (Supplementary Fig. 11) that ultimately fractured for high cycle number, as demonstrated by the observation of the bare flat Si/SiO_x_ for Si/SiO_x_/Ni/NiFePB-*150cy* (Supplementary Figs. 10d and 11d). Finally, transmission electron microscopy (TEM) was also performed on a thinned *p*^*+*^-Si/SiO_x_/Ni/NiFePB-*75cy* to visualize the junction. Figure 2a, and STEM-EDS analyses (Supplementary Fig. 13), confirmed the presence of the NiFePB layer (~35 nm) over the Ni thin film. High-resolution TEM (Fig. 2b) revealed the amorphous nature of the NiFePB, as well as the high Ni porosity, generated by the electrodissolution. It is also interesting to note that NiFePB interpenetrated the Ni layer, forming a robust interface. The SiO_x_ tunnel layer could also be observed between Si and Ni (Fig. 2c), and had a thickness of 1.9 nm, in close agreement with the ellipsometric value (Supplementary Fig. 1). To sum up, the combination of the characterization data demonstrates the conversion of the initially homogeneous Si/SiO_x_/Ni to a highly inhomogeneous Si/SiO_x_/Ni/NiFePB MIS junction. This structure, where the redox-active layer (NiFePB) is immobilized on the Ni thin film, should allow to directly probe the photoelectrochemistry of the junction in a much less harsher environment than the typical strongly alkaline solutions employed for OER or UOR, precluding Ni oxidation and enabling a precise evaluation of the effect of inhomogeneity on photovoltage.

### Electrochemistry and photoelectrochemistry in 1 M KCl

**Fig. 3:**
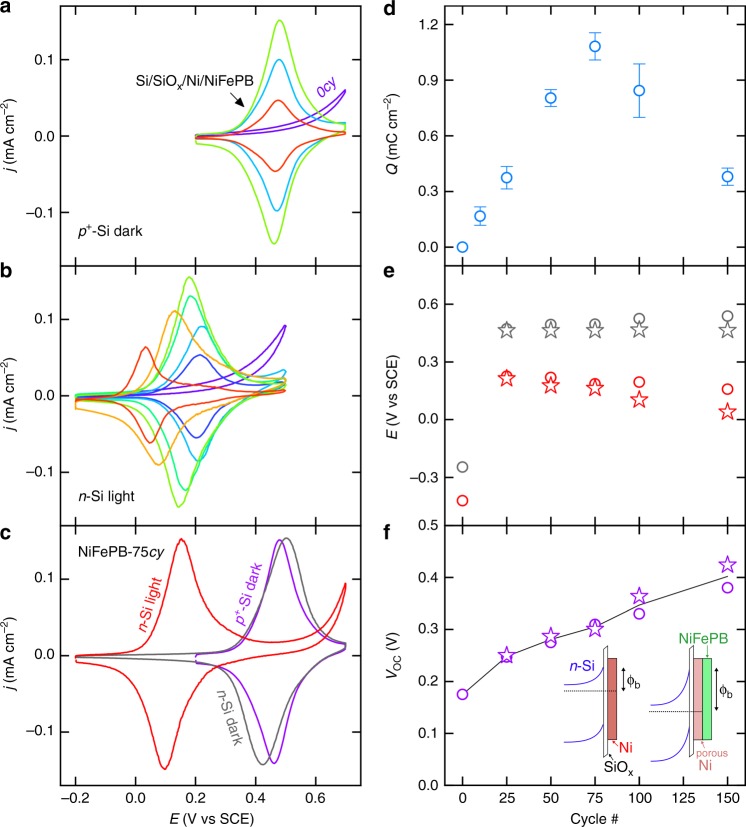
Electrochemistry and photoelectrochemistry of Si/SiO_x_/Ni/NiFePB anodes. **a**, **b** CVs recorded **a** in the dark on *p*^*+*^-Si/SiO_x_/Ni/NiFePB and **b** under simulated sunlight on *n*-Si/SiO_x_/Ni/NiFePB, prepared with different number of cycles ((purple) *0cy*, (dark blue) *10cy*, (light blue) *25cy*, (cyan) *50cy*, (light green) *75cy*, (yellow) *100cy*, and (red) *150cy*). **c** CVs recorded in the dark on (purple) *p*^*+*^-Si/SiO_x_/Ni/NiFePB-*75cy* and (gray) *n*-Si/SiO_x_/Ni/NiFePB-*75cy* and under simulated sunlight on (red) *n*-Si/SiO_x_/Ni/NiFePB-*75cy*. **d** Integrated average charge of the oxidation peak for Si/SiO_x_/Ni/NiFePB anodes as a function of the number of cycles (the errors bars represent the standard deviation of at least four independent measurements). **e** Values of (disks) OCP and (stars) *E*^0′^ measured in the dark (gray) and under simulated sunlight (red) on *n*-Si/SiO_x_/Ni/NiFePB as a function of the number of cycles. **f** Values of *V*_oc_ determined by (purple disks) OCP and (purple stars) *E*^0′^ measurements on *n*-Si/SiO_x_/Ni/NiFePB as a function of the number of cycles. The black curve represents the variation of the average value. Inset: schematic band diagrams of left) *n*-Si/SiO_x_/Ni and right) *n*-Si/SiO_x_/Ni/NiFePB junctions in the dark, showing the increasing of the barrier height (*ϕ*_b_) after modification. All measurements were performed in Ar-saturated 1 M KCl, the CVs were recorded at 10 mV s^−1^

The electrochemical activity of the *p*^*+*^-Si MIS anodes, recorded in the dark in 1 M KCl is first presented in Fig. 3a. Unlike the homogeneous *p*^*+*^-Si/SiO_x_/Ni anode (purple curve), the *p*^*+*^-Si/SiO_x_/Ni/NiFePB anodes exhibited a marked redox activity, as shown by the reversible wave that was observed for all modified electrodes. This wave corresponds to the Fe^III^/Fe^II^ redox couple in NiFePB, as supported by (i) the value of the apparent standard potential (*E*^0′^ = 0.47 V vs SCE, Supplementary Table 1), which is very close to that reported in the literature for similar Prussian blue systems^24^ as well as (ii) the linear relationship between the current density of the redox peaks and the scan rate (Supplementary Fig. 14) that demonstrates the immobilized nature of the redox system^27^. As it can be observed in Fig. 3b, the Fe^III^/Fe^II^ redox system was also observed for *n*-Si/SiO_x_/Ni/NiFePB photoanodes under illumination with simulated sunlight, though, at a lower *E*^0′^ value, which was dependent on the cycle number. In the dark, the redox waves were also observed (Supplementary Fig. 15) but at a *E*^0′^ that was constant for all *n*-Si/SiO_x_/Ni/NiFePB (*E*^0′^ = 0.46 V *vs* SCE, Supplementary Table 1) and statistically identical to that recorded on *p*^*+*^-Si/SiO_x_/Ni/NiFePB anodes. Figure 3c illustrates this phenomenon for Si/SiO_x_/Ni/NiFePB-*75cy*, which suggests that the dark oxidation of the NiFePB coating is achieved through *e*^−^ injection into the Si conduction band, as opposed to the oxidation under illumination, which occurs through the injection of photogenerated *h*^*+*^ from the Si valence band to the NiFePB (Supplementary Fig. 16). It can be clearly observed in Fig. 3b that the *E*^0′^ value decreased with an increasing number of cycles, implying a considerable improvement of the photovoltage (*V*_oc_). Note that the unconventional CV shape obtained for *n*-Si/SiO_x_/Ni/NiFePB-*150cy* under illumination, characterized by an inverted position of the oxidation and the reduction waves (the reduction wave occurs at a more positive potential than the oxidation wave), was reproducible and is attributed to the electrostatic interactions between the NiFePB layer and the charges in the space-charge layer^28^.

The anodic charge (*Q*) corresponding to the oxidation of Fe^II^ into Fe^III^ was extracted from the CVs and studied as a function of the number of cycles. While *Q* was found independent on the Si doping, a remarkable dependency with the cycle number was found (Fig. 3d). Indeed, *Q* increased linearly (due to the increase of the NiFePB layer thickness), up to a maximum value of 1.1 mC cm^−2^ at *75cy* (corresponding to a 1.1 × 10^−8^ mol cm^−2^ surface coverage in electroactive Fe centers), and decayed for a higher number of cycles. The decrease can be explained by the fracturing of the Ni thin film (that was evidenced for high cycle numbers by SEM, Supplementary Figs. 10d and 11d), which disrupts the charge conduction pathway between NiFePB and Si, thus lowering the number of electrochemically addressable Fe centers. The photovoltage (*V*_oc_) generated at the *n*-Si/SiO_x_/Ni/NiFePB surfaces was determined either from the open-circuit potentials (OCPs, Supplementary Fig. 17) or the *E*^0′^ values extracted from the CVs, both in the dark and under illumination in 1 M KCl, as shown in Fig. 3e. The resulting *V*_oc_ values are plotted in Fig. 3f as a function of the number of cycles. From this curve, it is clear that the modification improved *V*_oc_, that varied from 0.18 V for the unmodified, homogeneous *n*-Si/SiO_x_/Ni MIS electrode to 0.40 V for *n*-Si/SiO_x_/Ni/NiFePB-*150cy*. Additional OCP measurements were performed in electrolytes containing the dissolved Fe(CN)_6_^3−^ and Fe(CN)_6_^4−^ redox species at concentration ratios ranging from 10^−3^ to 10^3^ (Supplementary Fig. 18a, b). The resulting *V*_oc_ values were comprised between 0.27 to 0.32 V, a difference which is about seven times smaller than the 360 mV-shift of solution potential predicted by the Nernst equation. In addition, OCP measurements in equimolar solutions of the Fe(CN)_6_^3−^/Fe(CN)_6_^4−^ and Ru(NH_3_)^3+^/ Ru(NH_3_)^2+^ redox couples (Supplementary Fig. 18c) as well as measurements in an electrolyte saturated with Ar and O_2_ gases (Supplementary Fig. 18d), indicated, in all cases, *V*_oc_ values comprised between 0.29 and 0.32 V. These OCP measurements demonstrate that *V*_oc_ is independent of the solution potential and that the photovoltage is controlled by the solid interface. Based on this series of photoelectrochemical experiments and on the previously discussed characterization data, the increase of *V*_oc_ with the cycle number can be rationally explained by the dissolution of the Ni layer, which induces the formation of pores in the Ni metal thin film (Supplementary Figs. 10 and 11). The layer permeation disrupts the initially continuous Si/SiO_x_/Ni tunnel Schottky junction and generates Ni-deficient Si/SiO_x_ regions with a larger barrier height (*ϕ*_b_), thus improving the photoanode overall *V*_oc_ (Fig. 3f, inset). Such a behavior has been previously observed^20,22,29^ and discussed in the context of OER photoanodes^16^ as well as solid-liquid solar cells^30^ and the high *ϕ*_b_ in these Si/SiO_x_ regions is thought to originate from the unpinning of the Schottky junction or from the equilibration of the Si with defect states in the SiO_x_. To summarize, the photoelectrochemical experiments demonstrate that the electrodissolution method considerably improved the photovoltage of homogeneous *n*-Si/SiO_x_/Ni MIS electrodes by disrupting the Schottky junction. Now, we discuss their catalytic activity.

### Electrochemistry in 1 M KOH

**Fig. 4:**
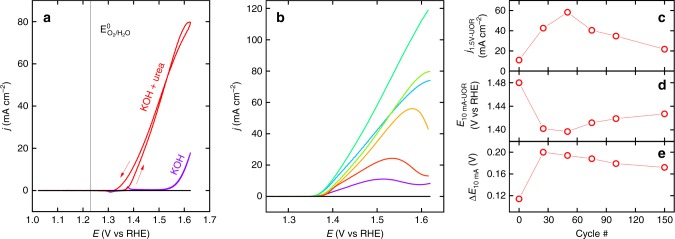
Electrocatalytic activity of *p*^*+*^-Si/SiO_x_/Ni/NiFePB anodes. **a** CVs of (black) *p*^*+*^-Si/SiO_x_ in 1 M KOH, (purple) *p*^*+*^-Si/SiO_x_/Ni/NiFePB-*75cy* in 1 M KOH and in (red) *p*^*+*^-Si/SiO_x_/Ni/NiFePB-*75cy* in 1 M KOH + 0.33 M urea, the arrows indicate the sweep direction. **b** LSVs of (black) *p*^*+*^-Si/SiO_x_ and *p*^*+*^-Si/SiO_x_/Ni/NiFePB, prepared with different number of cycles ((purple) *0cy*, (light blue) *25cy*, (cyan) *50cy*, (light green) *75cy*, (yellow) *100cy*, (red) *150cy*) in 1 M KOH + 0.33 M urea. **c–e** Values of **c** *j*_1.5V-UOR_, **d** *E*_10mA-UOR_, and **e** Δ*E*_10mA_ measured for *p*^*+*^-Si/SiO_x_/Ni/NiFePB as a function of the number of cycles. All voltammograms were recorded at 10 mV s^−1^ in the dark

In order to only assess the electrocatalytic activity of the Ni/NiFePB layers and to preclude light-induced phenomena, we studied the anodic behavior of the *p*^*+*^-Si/SiO_x_/Ni/NiFePB MIS anodes in the dark in 1 M KOH, with a particular focus on UOR. As illustrated by Fig. 4a, which shows the anodic behavior for *p*^*+*^-Si/SiO_x_/Ni/NiFePB-*75cy*, the anodes exhibited distinct electrochemical responses in the presence and in the absence of urea. It is also interesting to note the absence of electrochemical response of *p*^*+*^-Si/SiO_x_ in both media (black voltammograms of Fig. 4a, b) revealing the absolute necessity of using the Ni coating for promoting charge transfer. In the case of the *p*^*+*^-Si/SiO_x_/Ni/NiFePB electrodes, a quasi-reversible redox wave was observed in the absence of urea at ~1.37 V vs RHE (Supplementary Fig. 19a), attributed to the Ni^III^/Ni^II^ system (generally observed for the NiOOH/Ni(OH)_2_ redox couple in alkaline solutions)^31^, followed by OER at potentials > 1.5 V vs RHE (Supplementary Fig. 19b). In the presence of 0.33 M of urea (this concentration was used because it is the average concentration in human urine^7^ and also a benchmark in the UOR-related literature, Supplementary Table 2) a catalytic current appeared at the position of the Ni^III^/Ni^II^ oxidation wave, attributed to UOR, in good agreement with the literature^7–9^.

Figure 4b compares linear sweep voltammograms (LSVs) recorded on Si/SiO_x_ and Si/SiO_x_/Ni/NiFePB prepared with electrochemical cycles ranging from *0cy* to *150cy*, in the urea-containing electrolyte (CVs are presented in Supplementary Fig. 20). UOR was observed for all MIS anodes with a catalytic activity that was clearly dependent on the number of cycles. Figure 4c–e show respectively the benchmark values of current density at 1.5 V vs RHE (*j*_1.5V-UOR_), potential at *j* = 10 mA cm^−2^ (*E*_10mA_) in the presence of urea (*E*_10mA-UOR_), as well as the difference between the *E*_10mA_ values measured in the absence and in the presence of urea (Δ*E*_10mA_ = *E*_10mA-OER_ − *E*_10mA-UOR_). In other words, Δ*E*_10mA_ represents the gain of potential brought by UOR as a function of the number of cycles; while the lowest Δ*E*_10mA_ (0.11 V) was obtained for the homogeneous *p*^*+*^-Si/SiO_x_/Ni, the modified *p*^*+*^-Si/SiO_x_/Ni/NiFePB surfaces presented a Δ*E*_10mA_ comprised between 0.17 and 0.20 V (Fig. 4e). *E*_10mA-UOR_ represents the thermodynamic requirement to reach a benchmark catalytic *j* of 10 mA cm^−2^ and it can be observed that *E*_10mA-UOR_ was maximum for *p*^*+*^-Si/SiO_x_/Ni (1.48 V vs RHE) and significantly lower for the *p*^*+*^-Si/SiO_x_/Ni/NiFePB anodes (Fig. 4d), with a minimum value of 1.40 V vs RHE for *p*^*+*^-Si/SiO_x_/Ni/NiFePB-*50cy*. Interestingly, this anode also exhibited the highest *j*_1.5V-UOR_ values (Fig. 4c), which makes it the most active anode. Conversely, all benchmark values recorded for the homogeneous *p*^*+*^-Si/SiO_x_/Ni were the worst among all tested MIS anodes, making it the least active anode. These results show that the electrochemical dissolution method improves considerably UOR; next, we investigate the origin of this behavior.

### Characterization of the active phase

**Fig. 5:**
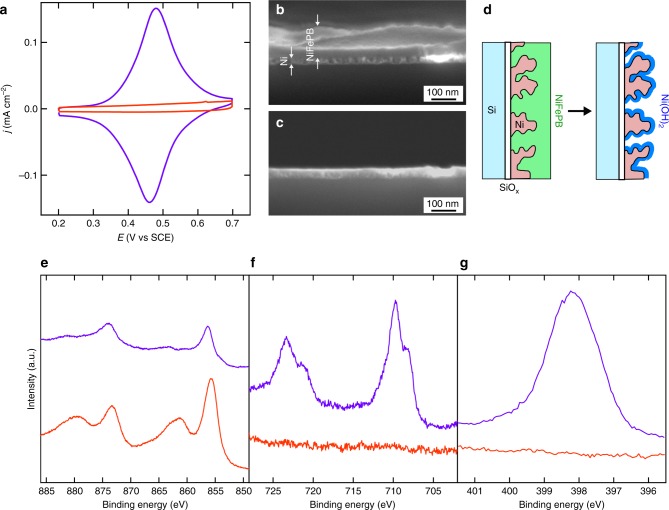
Characterization of the UOR-active phase. **a** CVs in 1 M KCl of *p*^*+*^-Si/SiO_x_/Ni/NiFePB*−75cy* at 10 mV s^−1^ in the dark (purple) after preparation and (red) after UOR. **b**, **c** Cross-section SEM images of *p*^*+*^-Si/SiO_x_/Ni/NiFePB-*75cy* **b** after preparation and **c** after UOR. **d** Scheme showing the changes occurring at a Si/SiO_x_/Ni/NiFePB surface during UOR. **e**–**g** XPS spectra showing **e** Ni 2p, **f** Fe 2p, and **g** N 1 s regions; for *p*^*+*^-Si/SiO_x_/Ni/NiFePB*-75cy* (purple) after preparation and (red) after UOR

In order to gain insights into both chemical and structural compositions of the UOR-active phase, *p*^*+*^-Si/SiO_x_/Ni/NiFePB-*75cy* surfaces were characterized before and after UOR by a series of analytical techniques, which all unveiled a drastic change of the surface under UOR conditions. First, CVs in 1 M KCl (Fig. 5a) showed the total inhibition of the Fe^III^/Fe^II^ redox wave (vide supra) after UOR. Second, XPS revealed a clear chemical evolution of the outermost interface. This appeared as a change in the composition of the Ni phase, indicated by an increase of the intensity of the Ni 2p peaks as well as a −0.6 eV-shift of the Ni 2p_3/2_ binding energy (Fig. 5e). In addition, XPS unveiled a total loss of the Fe (Fig. 5f) and N (Fig. 5g) contents (if Fe atoms are present in this layer, Fig. 5f indicates that their amount is below the XPS detection limit). These results are consistent with the decomposition of NiFePB into Ni(OH)_2_ and Fe hydroxides, the latter being simultaneously released in solution, a behavior that has been previously reported for Prussian blue derivatives immersed in highly alkaline media^24^. This is well in line with the Ni 2p spectrum obtained after UOR, which is strictly identical to that reported for Ni(OH)_2_^32^. Furthermore, SEM revealed considerable structural changes (Fig. 5b, c) and confirmed the removal of the NiFePB film during UOR (additional SEM images are shown in Supplementary Figs. 21 and 22). To sum up, the characterization results obtained after UOR demonstrate that, in these conditions, NiFePB reacts with KOH to generate a Ni(OH)_2_ phase on the Ni film, which is electrochemically converted to NiOOH at potentials more positive than 1.37 V vs RHE (Supplementary Fig. 19a)^31,33^. The considerable improvement in catalytic activity observed between the unmodified homogeneous Si/SiO_x_/Ni and the inhomogeneous Si/SiO_x_/Ni/NiFePB MIS electrodes (Fig. 4b–e) indicates a relationship between the UOR activity and the porous Ni(OH)_2_ phase that is converted, during UOR, to a dense area of highly active NiOOH catalytic sites^7–9^. The evolution of the catalytic activity is thus correlated with the amount of porous Ni(OH)_2_ on the surface, which, according to the SEM analysis (Supplementary Fig. 11) and the charge under the Ni^III^/Ni^II^ redox wave (Supplementary Fig. 19a), first increases with the number of cycles and then decreases for prolonged electrodissolution (i.e. for cycle number >50). Based on these results, clearly showing the conversion of NiFePB into Ni(OH)_2_ under the UOR conditions, we will now refer to the Si/SiO_x_/Ni/NiFePB-*#cy* surfaces that are used for UOR as Si/SiO_x_/Ni/Ni(OH)_2_-*#cy*.

### Photoelectrochemistry in 1 M KOH

**Fig. 6:**
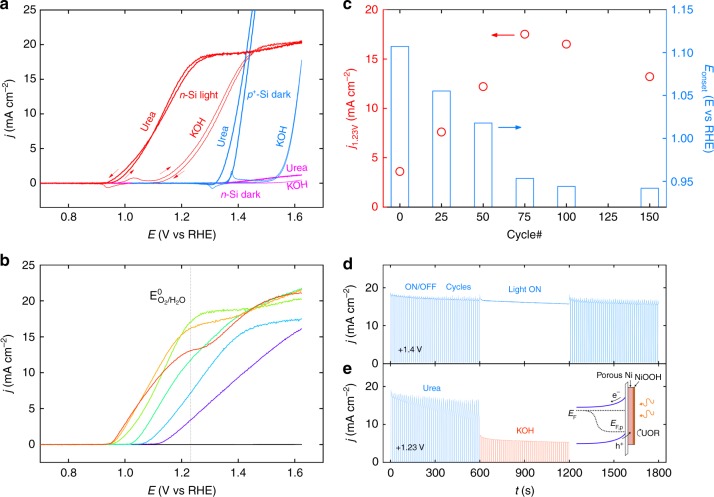
Photoelectrocatalysis on *n*-Si/SiO_x_/Ni/Ni(OH)_2_. **a** CVs of (light blue) *p*^*+*^-Si/SiO_x_/Ni/Ni(OH)_2_-*75cy* in the dark, (pink) *n*-Si/SiO_x_/Ni/Ni(OH)_2_-*75cy* in the dark and (red) *n*-Si/SiO_x_/Ni/Ni(OH)_2_-*75cy* under simulated sunlight; the CVs recorded in 1 M KOH are represented by thin lines and the CVs recorded in 0.33 M urea + 1 M KOH are represented by thick lines. **b** LSVs of (black) *n*-Si/SiO_x_ and *n*-Si/SiO_x_/Ni/Ni(OH)_2_, prepared with different number of cycles ((purple) *0cy*, (light blue) *25cy*, (cyan) *50cy*, (light green) *75cy*, (yellow) *100cy*, and (red) *150cy*) under simulated sunlight in 0.33 M urea + 1 M KOH. All voltammograms were recorded at 10 mV s^−1^. **c** Values of (red disks) *j*_1.23V_ and (blue bars) *E*_onset_ recorded under simulated sunlight in 0.33 M urea + 1 M KOH. **d**, **e** *j* vs *t* plots for UOR on *n*-Si/SiO_x_/Ni/Ni(OH)_2_-*75cy* recorded at 1.40 V (**d**) and 1.23 V (**e**) under intermittent and continuous light illumination in 0.33 M urea + 1 M KOH. In **e**, the electrolyte was replaced at 600 s by 1 M KOH. Inset: schematic band diagrams of a *n*-Si/SiO_x_/Ni/NiOOH photoanode in operation

Having addressed systematically the two beneficial effects of the electrodissolution method, namely, the improvement of the photovoltage at the MIS Schottky junction (Fig. 3) and the improvement of the UOR electrocatalysis on the metal thin film (Fig. 4), we now combine both effects by employing photoactive *n*-Si/SiO_x_/Ni/Ni(OH)_2_ for UOR. Figure 6a presents the CVs recorded on *n*-Si/SiO_x_/Ni/Ni(OH)_2_-*75cy* in 1 M KOH in the dark and under illumination, in the absence and in the presence of urea. The current densities obtained in the dark were very small (<1.5 mA cm^−2^), conversely, they became much higher under illumination (*j*_max_ = 20.6 mA cm^−2^), showing that photogenerated holes triggered OER and UOR at high rates (inset of Fig. 6e). Figure 6a also demonstrates the gain of electrical energy that occurs for both reactions when employing illuminated *n*-Si instead of non-photoactive *p*^*+*^-Si. The potential gain at the illuminated *n*-Si/SiO_x_/Ni/Ni(OH)_2_-*75cy* was 0.28 V at *j* = 10 mA cm^−2^ (for both OER and UOR, Supplementary Fig. 23), in good agreement with the *V*_oc_ previously measured on *n*-Si/SiO_x_/Ni/NiFePB-*75cy* (0.30 V, Fig. 3f). Additional OCP measurements performed in an aqueous electrolyte containing dissolved Fe(CN)_6_^3−^ and Fe(CN)_6_^4−^on *n*-Si/SiO_x_/Ni/NiFePB-*75cy* before and after UOR (Supplementary Fig. 24) revealed, in both cases a *V*_oc_ value of 0.30 V, showing that the removal of the NiFePB layer did not influence *V*_oc_. This confirms that the photovoltage is, in any case, controlled by the inhomogeneous Si/SiO_x_/Ni junction and that the electrolyte-permeable NiFePB and Ni(OH)_2_ layers do not contribute to *V*_oc_.

In addition to this gain caused by the illuminated MIS junction, UOR further shifts negatively the voltammograms with respect to OER, by Δ*E*_10mA_ = 0.19 V on *n*-Si/SiO_x_/Ni/Ni(OH)_2_-*75cy*. This value is in perfect agreement with that measured on non-photoactive *p*^*+*^-Si/SiO_x_/Ni/Ni(OH)_2_-*75cy* (Fig. 4a) confirming that this gain is entirely induced by UOR. The combination of both phenomena allows generating an overall thermodynamic gain of 0.47 V, leading to *E*_10mA_ = 1.13 V vs RHE for *n*-Si/SiO_x_/Ni/Ni(OH)_2_-*75cy* in the presence of urea. Remarkably, note that, in these conditions, the photocurrent plateau is almost reached at the standard potential of the O_2_/H_2_O couple, which is something normally only obtained with Si photoanodes bearing a buried *np*^+^ junction^34–36^ clearly demonstrating the importance of electrolyte composition for improving the performance of the electrodes and saving energy for H_2_ production in PECs. Figure 6b shows the LSVs recorded under illumination in the presence of urea on *n*-Si/SiO_x_ and *n*-Si/SiO_x_/Ni/Ni(OH)_2_ surfaces modified with 0 to 150 cycles (corresponding CVs are shown in Supplementary Fig. 25). The voltammogram shape is quite indicative of the processes occurring at the illuminated photoanode, with a slope reflecting the reaction kinetics. Indeed, as demonstrated for *p*^*+*^-Si/SiO_x_/Ni/Ni(OH)_2_ (Fig. 4b), UOR is much slower on the unmodified surface (*n*-Si/SiO_x_/Ni/Ni(OH)_2_-*0cy)* with respect to the modified ones, which is also observed for the photoactive surfaces (Fig. 6b). In addition, the appearance of a shoulder, observed for the highest cycle numbers (*n*-Si/SiO_x_/Ni/Ni(OH)_2_-*100cy* and *150cy*) indicates a decrease of UOR activity, which is supported by a larger OER photocurrent density at higher potentials. Because of the photovoltage enhancement (vide supra), the UOR onset potentials (*E*_onset_, arbitrarily set for *j* = 100 μA cm^−2^) shifted negatively with the cycle number, varying from 1.11 V vs RHE for the homogeneous MIS *n*-Si/SiO_x_/Ni/Ni(OH)_2_-*0cy* to 0.94 V vs RHE for *n*-Si/SiO_x_/Ni/Ni(OH)_2_-*150cy* (Fig. 6c and S26), which is, in any case, lower than what has been reported on conducting catalytic electrodes^7–9^. While at low cycle numbers (*0cy* to *75cy*), the increase of *V*_oc_ and the improvement of the UOR catalysis are cumulative and both contribute to a better overall performance, for high cycle numbers (>*75cy*) the catalysis activity decays because both effects become antagonist, decreasing the *j* value at *E*^0^_O2/H2O_ (*j*_1.23V_, Fig. 6c). In addition, the CV analysis reveals that high cycle numbers are also detrimental to the photoanode stability (Supplementary Fig. 27). We have thus selected for the following *n*-Si/SiO_x_/Ni/Ni(OH)_2_-*75cy*, which represents the best compromise in terms of activity and stability. From Mott–Schottky (M–S) measurements performed on this electrode, a carrier density of 4 × 10^16^ cm^−3^ as well as an effective barrier height *ϕ*_b_ = 0.84 eV was determined, enabling to build the band diagram of the interface (Supplementary Fig. 28 and details in the Methods section). This value is consistent with previous reports on MIS photoanodes^19,20^ and considerably higher than that measured for the homogeneous *n*-Si/SiO_x_/Ni/Ni(OH)_2_-*0cy*, (*ϕ*_b_ = 0.62 eV), which further confirm our hypothesis of a higher band bending caused by the rupture of the Ni thin film upon electrodissolution (Supplementary Fig. 28b, c). The stability of these photoanodes was tested upon prolonged electrolysis. Figure 6d shows a chronoamperometry curve obtained at 1.4 V vs RHE, in the presence of urea under intermittent light. The current density varied instantaneously upon dark–light cycles and the photocurrent decayed only by 13% after 30 min (Supplementary Fig. 29a). In Fig. 6e, a similar anode was tested at 1.23 V vs RHE and after 10 min the electrolyte was replaced by a urea-free solution, which induced a ~3-fold drop of *j* with respect to the initial value (Supplementary Fig. 29b), clearly showing the benefit of UOR for improving the photoanode performance at low overpotentials.

## Discussion

In conclusion, we have reported that electrodissolution can be employed on homogeneous Si/SiO_x_/Ni MIS electrodes for improving the intrinsic photoelectrochemical properties of the junction. In addition, we have shown how a redox species can be immobilized on the surface during the dissolution process and how it can be employed to directly probe the photoelectrochemical properties of the junction. This method allowed us to unveil the remarkable photovoltage improvement caused by the etching of the initially homogeneous Ni thin film. A second important effect of the electrodissolution is the increase of the surface density of catalytically active Ni sites allowing for a considerable improvement of urea oxidation reaction (UOR). Both phenomena were studied independently and then combined to prepare highly active photoanodes for solar-assisted UOR. Our results clearly highlight the benefits of inhomogeneity in MIS photoelectrodes and we believe that this concept could lead to further improvements by further engineering of several parameters such as the SiO_x_ layer. In addition, we introduced UOR as a promising Si-compatible anodic reaction for improving the utility as well as the yield of PECs. Although issues related to catalyst deactivation (that could be improved by catalyst optimization)^9^ and compatibility with pure urine still have to be solved to enable real application of such systems, these findings open new doors for the design of artificial leaves, based on abundant materials, which can produce H_2_ with polluted water under sunlight.

## Methods

### Reagents

Acetone (MOS electronic grade, Erbatron from Carlo Erba) and anhydrous ethanol (RSE electronic grade, Erbatron from Carlo Erba) were used without further purification. The ultrapure water had a resistivity of 18.2 MΩ cm (Purelab Classic UV). Sulfuric acid (96%, VLSI grade Selectipur) and hydrogen peroxide (30%, VLSI, Sigma-Aldrich) were purchased from BASF and Sigma Aldrich, respectively. KOH (pellets for analysis) was purchased from Merck, KCl (99%) was purchased from Acros. Urea, K_3_Fe(CN)_6_ and K_4_Fe(CN)_6_ were purchased from Sigma Aldrich.

### Surface preparation

All vials and tweezers used for cleaning of silicon were previously decontaminated in 3/1 v/v concentrated H_2_SO_4_/30% H_2_O_2_ at 105 °C for 30 min, followed by copious rinsing with ultrapure water. Caution: the concentrated aqueous H_2_SO_4_/H_2_O_2_ (piranha) solution is very dangerous, particularly in contact with organic materials, and should be handled extremely carefully. The *n-*type silicon (0.3–0.7 Ω cm resistivity, phosphorus-doped, double side polished, 275–325 μm) (100) and *p*^*+*^-type silicon wafers (0.001 Ω cm resistivity, boron-doped, single side polished 275–325 μm) (100) were purchased from Siltronix. All the Si surfaces were degreased by sonication in acetone, ethanol, and ultrapure water for 10 min respectively. The surfaces were then decontaminated and oxidized in piranha solution at 105 °C for 30 min, followed by rinsing with copious amounts of ultrapure water. FTO-coated glass slides were purchased from Sigma Aldrich. The FTO slides were cleaned by sonication in LGC-300 (Transene), acetone, ethanol, and ultrapure water for 10 min respectively and dried under Ar flow.

### Ni thin film deposition

The 25-nm-thick Ni thin films were deposited on the clean FTO or Si/SiO_x_ surfaces by sputtering with a Leica EM ACE600 coating system (Ni target purity: 99.8%, Leica). The Ar pressure for sputtering was 2 × 10^−2^ mbar and the current 100 mA (pre-sputtering time of 1 min), the thickness of the film was determined in situ by a quartz crystal microbalance. After deposition, the system was degassed with N_2_.

### Electrode fabrication

The coated surfaces were further processed to fabricate electrodes. For FTO/Ni, an electrical contact with a metal wire was created on the top of the conducting slide using silver paste (Radiospares) and copper tape (Radiospares) and the electroactive area was defined using hydrophobic tape (3 M). For Si/SiO_x_/Ni, the Ohmic contact was done on the backside of Si wafer by scratching the surface with a diamond glass cutter; then a droplet of InGa eutectic and a metal wire were applied on the scratched part. A thin layer of silver paste was painted to cover the InGa eutectic contact as well as a part of the metal wire. After drying of the paste, epoxy resin (Loctite 9460, Henkel) was deposited to shield the backside and frontside of the surface except an active area comprised between 0.1 and 0.25 cm^2^, note that the exact geometrical value was precisely measured using the ImageJ software prior to the (photo)electrochemical experiments. The electrodes were baked in the oven at 90 °C for 1.5 h to cure the epoxy resin.

### Preparation of the NiFePB coatings

The electrodes were electrochemically modified with NiFePB using a freshly-prepared 5 mM K_3_Fe(CN)_6_ electrolyte (pH adjusted to 2.5 with concentrated HCl). We have tested different electrochemical methods that gave satisfactory results in terms of modification, however, the pulse method was found to give the deposit with the highest catalytic activity for urea oxidation. The electrodes were modified in a two electrode setup using a SP-150 Bio-Logic potentiostat. The Ni-coated electrode was employed as the anode and a large Pt mesh electrode as the cathode in a beaker filled with the electrolyte. The Pt mesh was placed on the beaker wall and the anode was placed in front of the counter electrode and sequential oxidation pulses of 1 s at 0.67 mA cm^−2^ were imposed followed by resting time of 3 s at 0 mA cm^−2^. The FTO and the *p*^*+*^-Si substrates were modified in the dark and the *n*-Si substrates were modified under illumination through the Pt mesh counter electrode with an optical fiber (Fiber Light DC950H from Dolan Jenner) equipped with a 150-W quartz halogen lamp. After the desired number of cycles was imposed, the electrode was removed from the cell, copiously rinsed with water and dried under an Ar stream.

### Electrochemical and photoelectrochemical experiments

Cyclic voltammetry (CV), chronoamperometry (CA), and open-circuit potential (OCP) measurements were performed in a three-neck cell comprising a quartz window and gas inlets. The reference electrodes were a Hg/HgO (1 M KOH) electrode for the measurements in alkaline solution and a KCl-saturated calomel electrode (SCE) for the measurements in 1 M KCl. The counter electrode was a graphite rod. The cell was filled with the electrolyte that was degassed with Ar gas before the experiments. The working electrode was placed in front of the quartz window. The light was provided by a solar simulator (LS0106, LOT Quantum Design) equipped with an AM 1.5 G filter. The power density of the light source was measured prior to experiments at the position of the photoelectrode using an ILT1400 radiometer (International Light Technologies) to ensure the right power density (100 mW cm^−2^). Electrochemical measurements were performed with a Zennium potentiostat (Zahner). The potentials versus Hg/HgO (1 M KOH) were converted into potentials versus reversible hydrogen electrode (RHE) using the following relation:

4 $$E_{{\mathrm{RHE}}} = E_{{\mathrm{Hg}}/{\mathrm{HgO}}} + 0.098 + 0.059{\mathrm{pH}} = E_{{\mathrm{Hg}}/{\mathrm{HgO}}} + 0.924$$

All reported potentials were intentionally not corrected by the Ohmic drop. Unless specified, the CVs and LSVs reported in this work were recorded at 10 mV s^−1^. The first CV scans were employed to produce Figs. 4 and 6. Impedance and OCP measurements were performed in Ar-degassed solutions of 1 M KCl or of K_3_Fe(CN)_6_/K_4_Fe(CN)_6_ in 0.1 M KCl. The impedance measurements were fitted using an equivalent circuit comprising a resistance (the cell resistance) in series with a resistance and a capacitance in parallel (simulating SiO_x_) in series with a resistance and a capacitance in parallel (simulating the semiconductor). The Mott–Schottky (M–S) experiments were performed using a graphite counter electrode and a Pt wire reference electrode in 0.5 mM/0.5 mM K_3_Fe(CN)_6_/K_4_Fe(CN)_6_ in 1 M KCl^37^. The cell was in the dark and the potential was swept from positive to negative values with a frequency of 10 kHz, an amplitude of 10 mV and 300 counts per point. The capacitance values were calculated using the previously-described equivalent circuit. The M–S equation relates the space-charge capacitance (*C*_SC_) to the applied voltage (*E*) across a semiconductor-electrolyte junction following:

5 $$\frac{1}{{C_{{\mathrm{sc}}}^2}} = \frac{2}{{qN_DA^2\varepsilon _0\varepsilon _r}}\left(E - E_{{\mathrm{fb}}} - \frac{{kT}}{q} \right)$$

where, *ε*_*r*_ is the relative permittivity of the semiconductor, *ε*_0_ is permittivity in vacuum, *A* is the surface area, *q* is the unsigned elementary charge, *N*_*D*_ is the density of free carriers, *k* is Boltzmann constant, and *T* is the temperature. The x-intercept of the linear region of the MS plot is indicative of the flatband potential *E*_fb_. In order to check the validity of the curves obtained in Supplementary Fig. 28a, we have calculated the doping density based on the following relation, obtained by substitution of the M–S equation:

6 $$N_{\mathrm{d}}({\mathrm{cm}}^{ - 3}) = \frac{{1.2 \times 10^{31}({\mathrm{cm}} \times F^{ - 2} \times V^{ - 1})}}{{{\mathrm{slope}}(F^{ - 2} \times {\mathrm{cm}}^4 \, \times V^{ - 1})}}$$

The slope of *n*-Si/SiO_x_/Ni/Ni(OH)_2_-*75cy* (Supplementary Fig. 28a), gives a *N*_d_ value of 4 × 10^16^ cm^−3^, which is relatively in good agreement with the resistivity value given by the wafer manufacturer (0.3–0.7 Ω cm). The barrier height (*ϕ*_b_), was then calculated using the following relation:

7 $$\phi_{\mathrm{b}} = - qE_{{\mathrm{fb}}} + V_{\mathrm{n}}$$

With *V*_n_ being the energy difference between the conduction band and the Fermi level, that was calculated using the following equation:

8 $$V_{\mathrm{n}} = kT\;{\mathrm{ln}}\left(\frac{{N_{\mathrm{c}}}}{{N_{\mathrm{d}}}} \right)$$

*N*_c_, the density of states in the conduction band was calculated by:

9 $$N_{\mathrm{c}} = 2\left( {\frac{{2\pi m_e^ \ast kT}}{{h^2}}} \right)^{3/2}$$

With *m*_e_* being the effective mass of electron in crystalline Si (9.389 × 10^−31^ kg) and *h* the Planck constant. The above equations allowed to calculate a *V*_n_ value of 0.15 eV. Thus, using the intersect of the linear fit of the M–S curves shown in Supplementary Fig. 28 values of 0.84 eV and 0.62 eV were calculated for *n*-Si/SiO_x_/Ni/Ni(OH)_2_-*75cy* and *n*-Si/SiO_x_/Ni/Ni(OH)_2_-*0cy*, respectively.

### Surface characterization

Scanning electron microscopy (SEM) was performed using a JSM 7100 F (JEOL). The sample was prepared for transmission electron microscopy (TEM) analysis as follows. First, the sample was cut in half using a dicing saw fitted with a diamond wire (Precision Diamond Wire Saw, Well 3241, *Escil*). The two fragments were then glued (G1 epoxy resin with hardener, *Gatan*) as follows: Si/SiO_x_/NiFePB/glue/NiFePB /SiO_x_/Si. This mounting was then cut to fit the TEM-slot (*Leica*) using a dicing saw. It was then glued onto the slot with the epoxy resin. The slot was then fixed onto a tripod using a mounting adhesive (crystal bond 509, cat#50400–01, Electron Microscopy Sciences) then polished (on both sides) using a sequence of polyamide/diamond papers with a range of particle sizes (from 30 to 1 μm). Finally, a felt polishing pad with colloidal silica was applied, with a final thickness for the area of interest of 20 μm. The slot was further thinned using dimpling grinder (Model 200, Fischione instruments) down to a 10-μm-thick target area before being further processed onto an ion milling machine (low angle ion milling and polishing system, Model 1010, Fischione instruments) to reach a final thickness of <100 nm for TEM analysis. TEM and scanning transmission electron microscopy (STEM) were performed with a JEM 2100 LaB_6_ (JEOL) equipped with a Silicon Drift Detector (SDD) - X-Max (Oxford Instruments) and the AZtec software to perform energy-dispersive X-ray spectroscopy and elemental mapping. XPS measurements were performed with an Mg K_alpha_ (hν = 1254.6 eV) X-ray source and an Al source (hν = 1486.6 eV) using a VSW HA100 photoelectron spectrometer with a hemispherical photoelectron analyzer, working at an energy pass of 20 eV for survey and resolved spectra. The experimental resolution was 1.0 eV. Unless specified, C1s set at 284.8 eV was used as the energy reference for all the analyses. Transmittance spectra were acquired on a Shimadzu UV-3600Plus photometer with an integrating sphere (ISR-603). Natural air atmosphere was used as 100% transmittance reference. Fourier transform infrared (FTIR) measurements were recorded in the grazing angle reflectance mode (A 513 accessory from Brüker Optics, 70° angle, 100 scans, 2 cm^−1^ resolution) using a Brüker Optics Vertex 70 FT-IR spectrometer equipped with a sensitive liquid-nitrogen-cooled MCT photovoltaic detector. The FTIR spectrum of Fig. 1c represents only the CN stretching region, as several measurements revealed that the other regions of the spectrum were not reproducible (probably due to the very low layer thickness). In order to get a higher signal/noise ratio, the *p*^*+*^-Si/SiO_x_ sample used for this experiment had a Ni layer of 50 nm and was modified with 150 electrochemical cycles. The cathodic polarization was performed at +0.2 V vs SCE for 30 s in the dark in 1 M KCl, the surface was then rinsed with ultrapure water and dried with Ar. The spectra were manually subtracted from the background (i.e., the spectrum recorded under the same conditions on a Ni-free Si/SiO_x_ surface) and the intensities of the main bands were normalized.

## Supplementary information

Peer Review File

Supplementary information

## Data Availability

All experimental data within the article and its Supplementary Information are available from the corresponding author upon reasonable request.
