# Supplementary material for: Tailoring the photoelectrochemistry of catalytic metal-insulator-semiconductor (MIS) photoanodes by a dissolution method
Source: Nat Commun. 2019 Aug 6;10:3522. doi: 10.1038/s41467-019-11432-1 (PMC6684633; doi:10.1038/s41467-019-11432-1)
Supplement: Supplementary file 2 — Supplementary information [file 41467_2019_11432_MOESM2_ESM.docx]

**Tailoring the photoelectrochemistry of catalytic
metal-insulator-semiconductor (MIS) photoanodes by a dissolution method**

G. Loget,*^1^ C. Meriadec,^2^ V. Dorcet,^1^ B. Fabre,^1^ A. Vacher,^1^ S. Fryars,^1^ S. Ababou-Girard^2^

^1^Univ Rennes, CNRS, ISCR (Institut des Sciences Chimiques de Rennes)-UMR6226 - ScanMAT-UMS2001, F-35000 Rennes, France. [gabriel.loget@univ-rennes1.fr](mailto:gabriel.loget@univ-rennes1.fr)

^2^Univ Rennes, CNRS, IPR (Institut de Physique de Rennes)-UMR 6251, F-35000 Rennes, France.

# Supplementary Figures

**
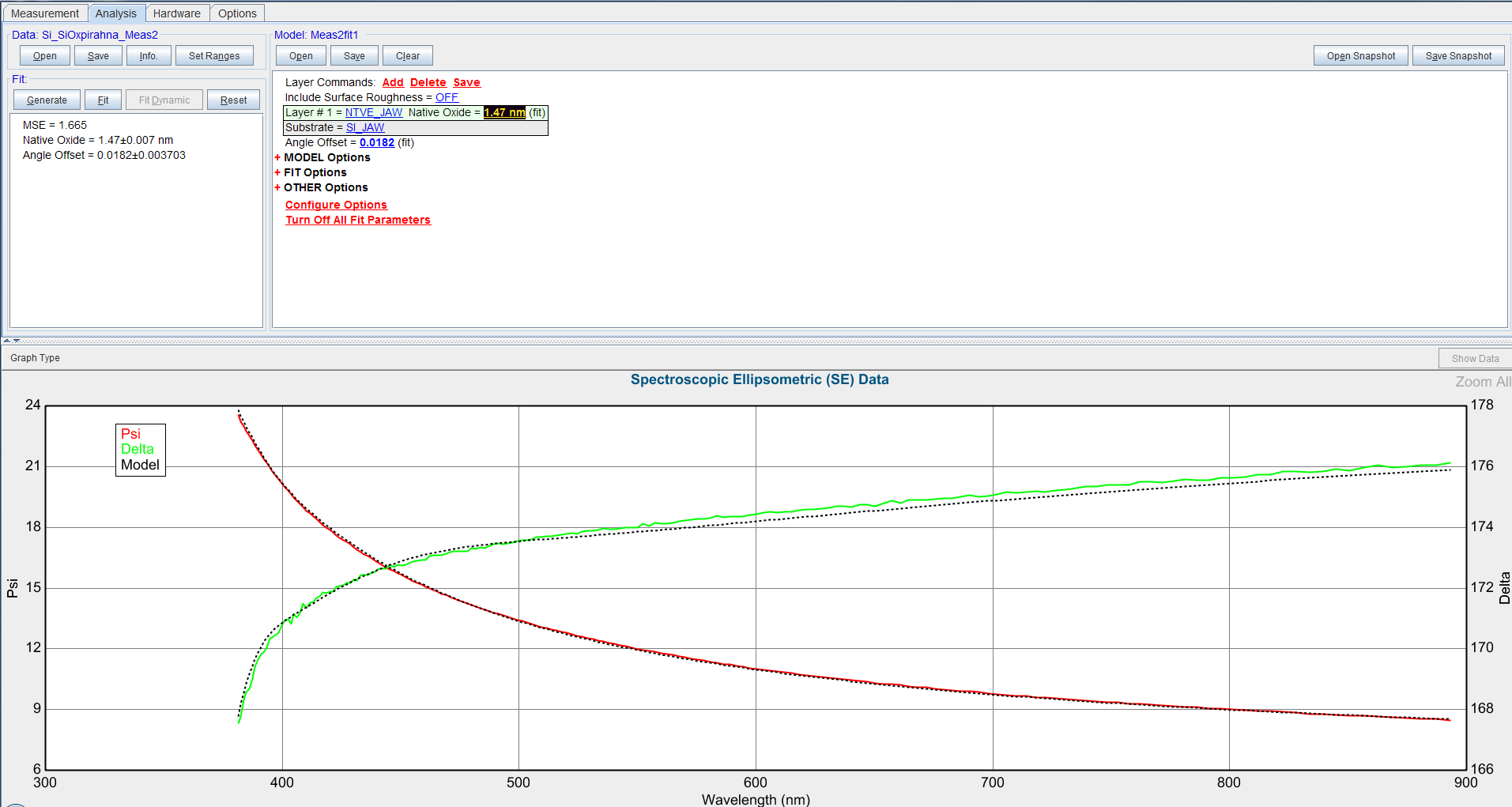
**

**Supplementary Figure 1. Ellipsometry on Si/SiO_x_.** Ellipsometry determination of the thickness of the SiO_x_ layer obtained after chemical oxidation with piranha solution (1/3 v/v H_2_O_2_ 30%/conc. H_2_SO_4_) at 105°C for 30 min.

**
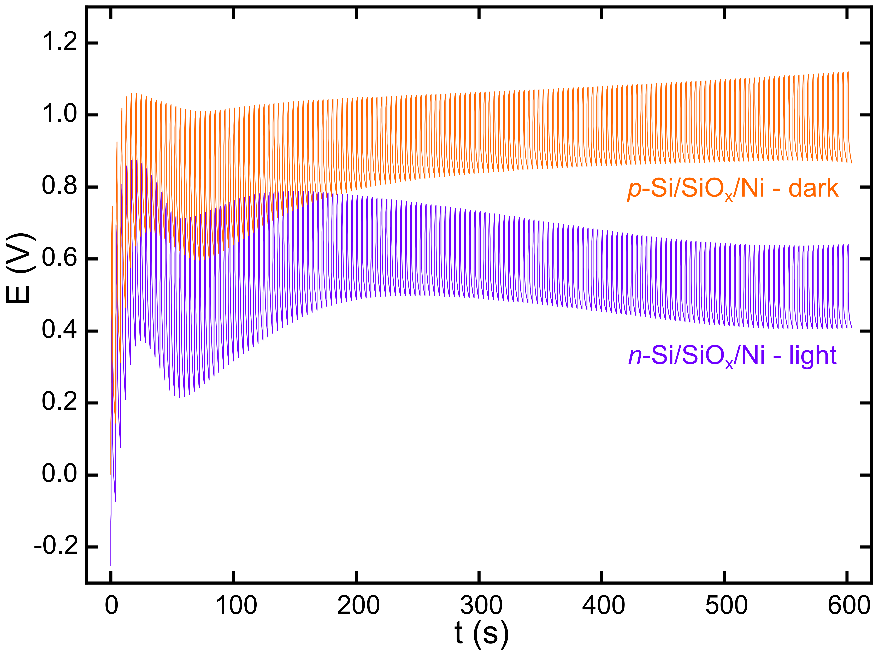
**

**Supplementary Figure 2. Dissolution of the Ni layer and formation of NiFePB.**  Chronopotentiometry curves obtained for the modification with NiFePB (150 cycles) of a *p^+^-*Si/SiO_x_/Ni electrode in the dark (orange curve) and an *n*-Si/SiO_x_/Ni electrode under illumination (violet curve).


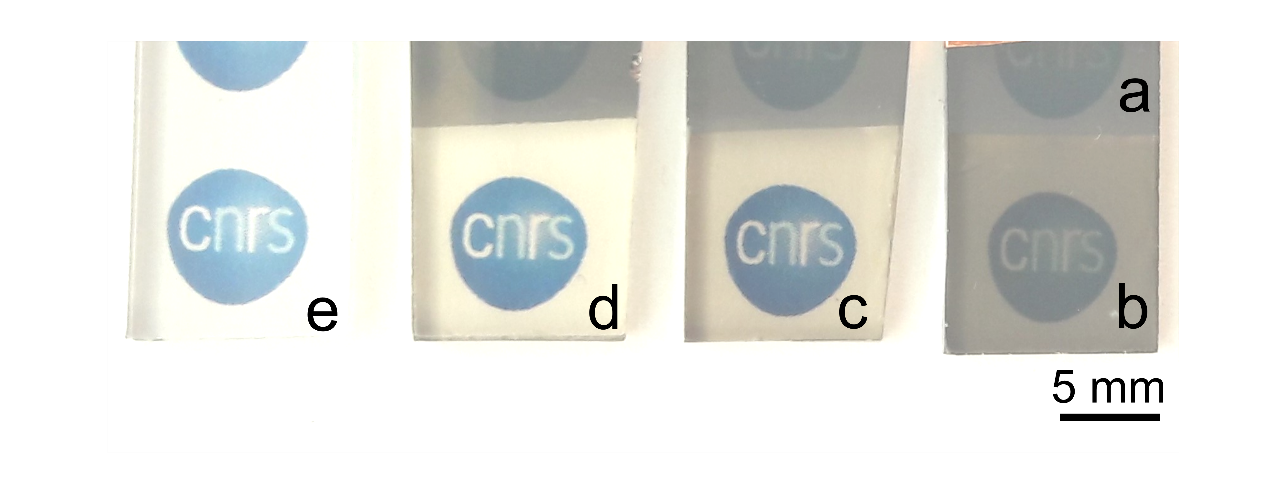


**Supplementary Figure 3.** **Photographs of FTO/Ni/NiFePB surfaces**. a) 0cy, b) 25cy, c) 75cy, d) 150cy and e) bare FTO.


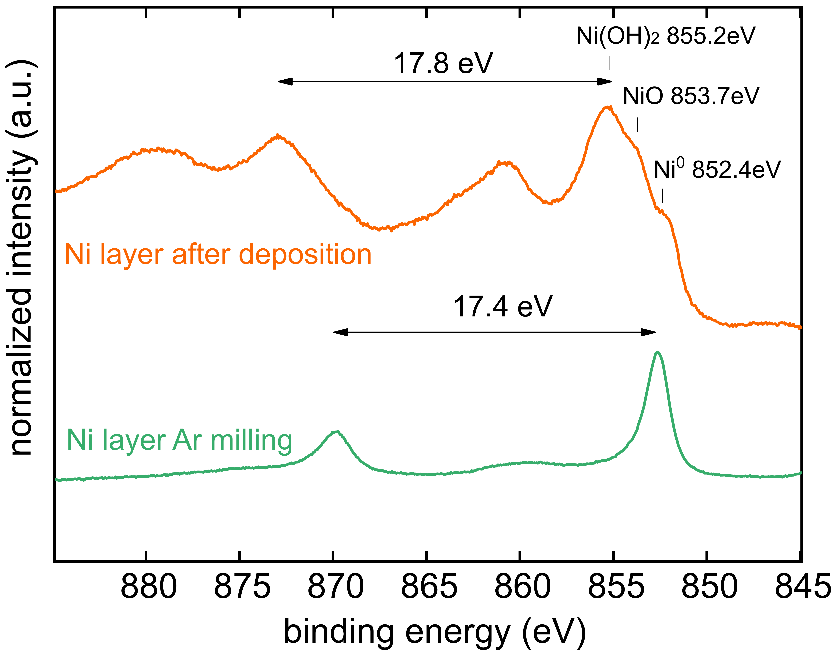


**Supplementary Figure 4. Ni milling experiments.** XPS spectra showing the Ni 2p region for *p^+^-*Si/SiO_x_/Ni-*0cy*: (top orange curve) the surface after sputtering of the Ni film and (bottom green curve) after Ar milling of the sputtered Ni film in the XPS chamber. The experimentally determined values of the Ni 2p_3/2_ binding energies for Ni^0^, NiO, and Ni(OH)_2_ are indicated on the top of the orange spectrum.


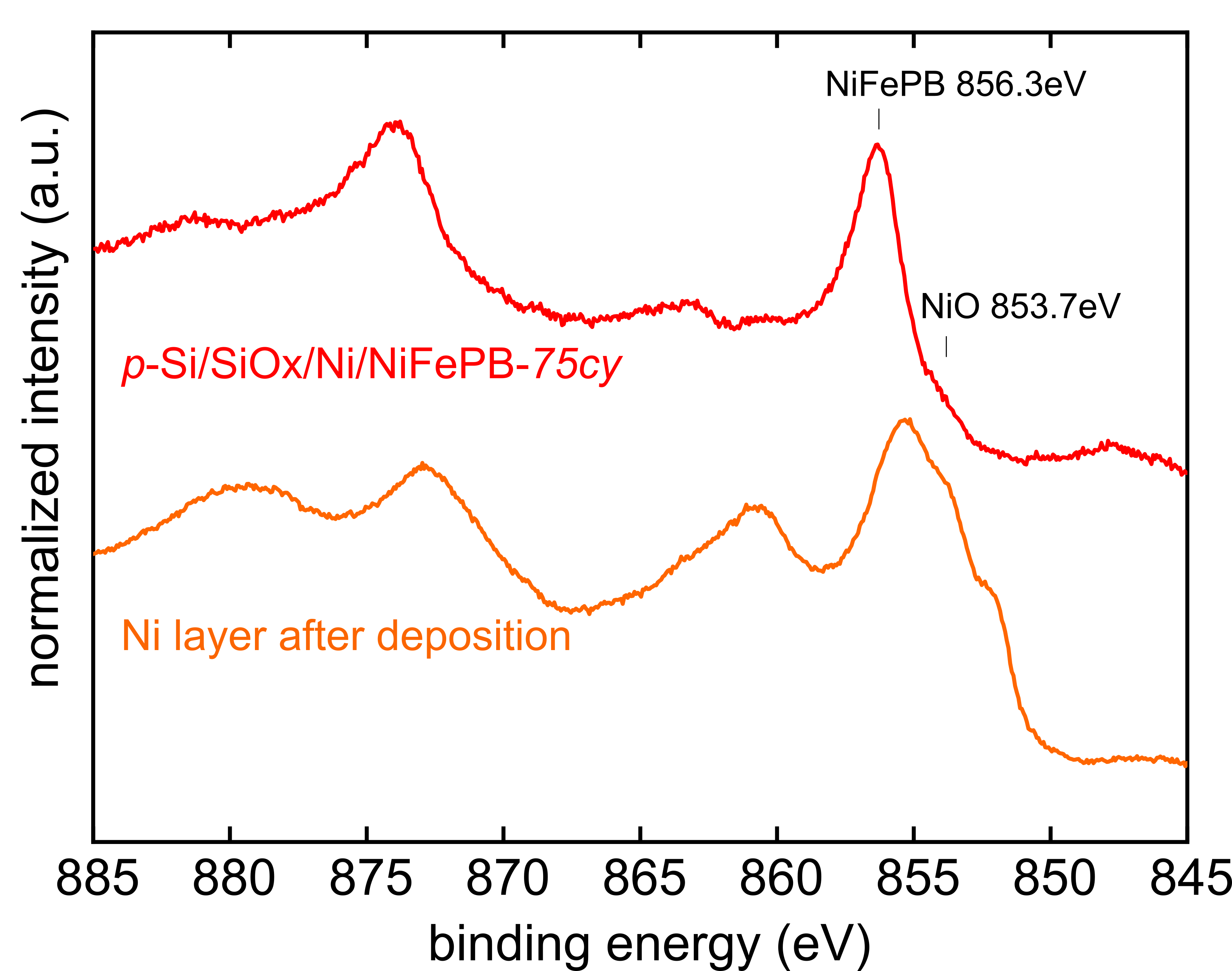


**Supplementary Figure 5. Ni investigation by XPS.** XPS spectra for (bottom orange curve) the electrode before modification (*p^+^-*Si/SiO_x_/Ni/NiFePB-*0cy*) and (top red curve) *p^+^-*Si/SiO_x_/Ni/NiFePB-*75cy*. The experimentally determined values of the Ni 2p_3/2_ binding energies for NiO and NiFePB are indicated on the top of the red spectrum.


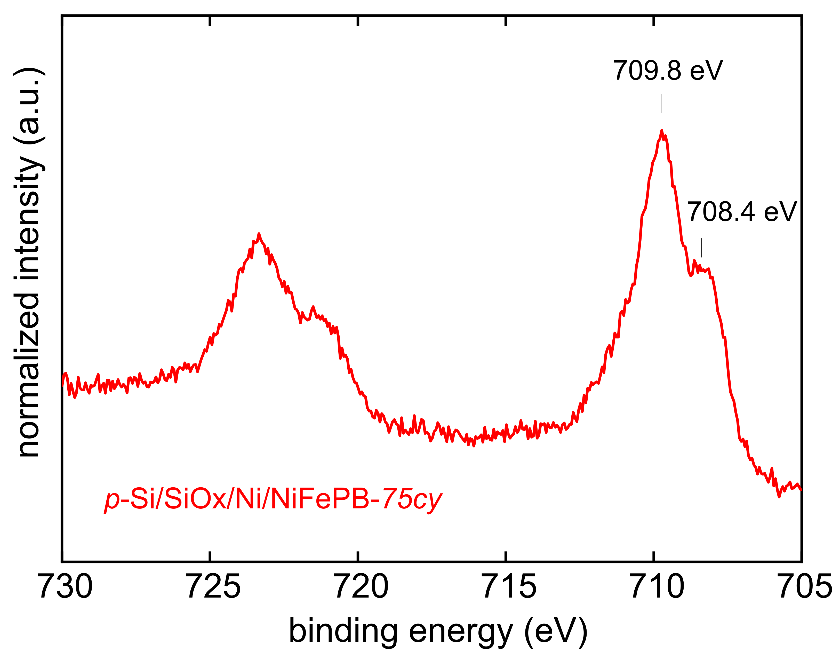


**Supplementary Figure 6.** **Fe investigation by XPS.** XPS spectrum showing the Fe 2p region for *p^+^-*Si/SiO_x_/Ni/NiFePB-*75cy*.


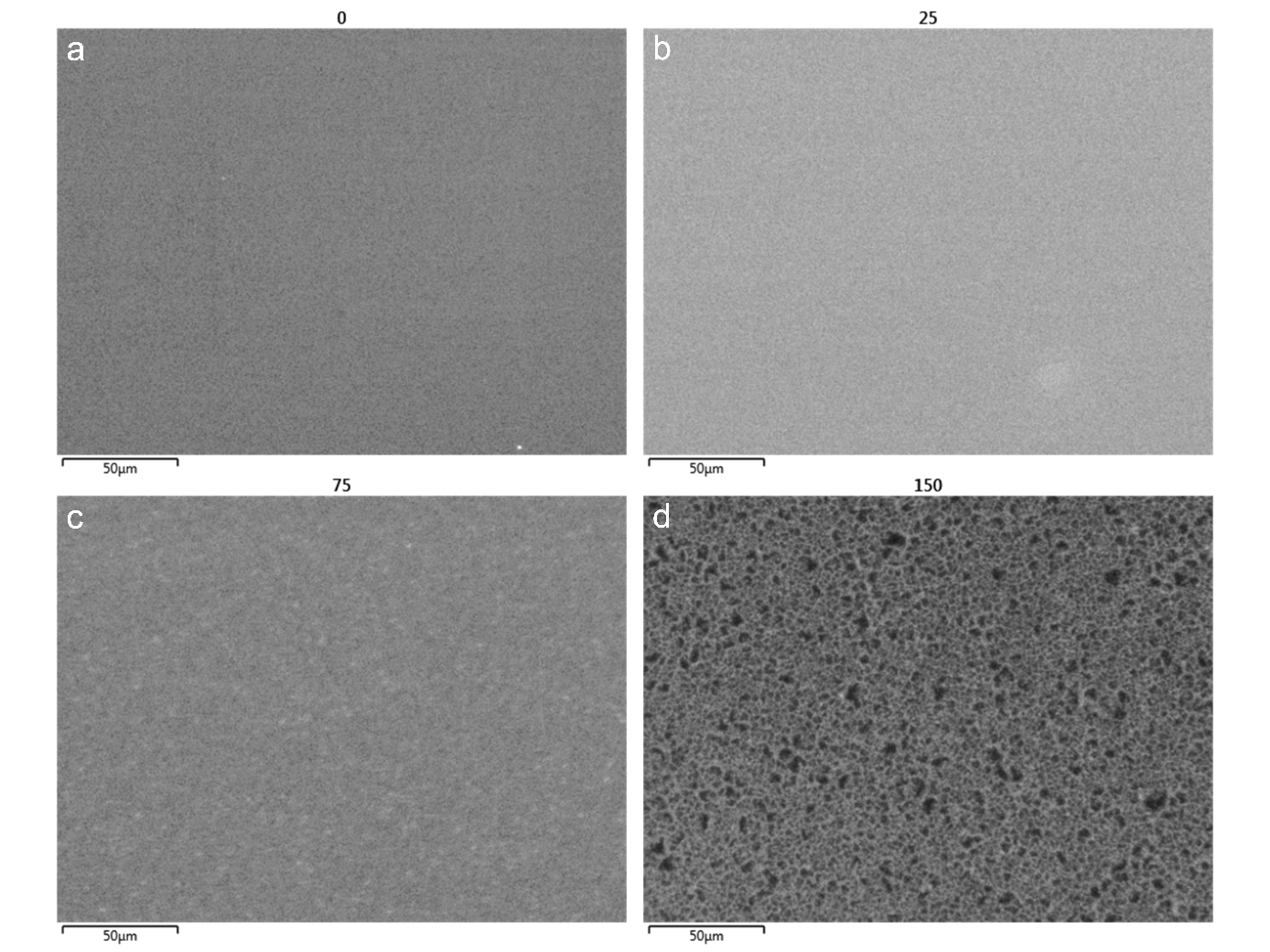


**Supplementary Figure 7. SEM images of the areas analyzed by EDS.** a) *p^+^-*Si/SiO_x_/Ni/NiFePB-*0cy*, b) *p^+^-*Si/SiO_x_/Ni/NiFePB-*25cy*, c) *p^+^-*Si/SiO_x_/Ni/NiFePB-*75cy* and d) *p^+^-*Si/SiO_x_/Ni/NiFePB-*150cy*.


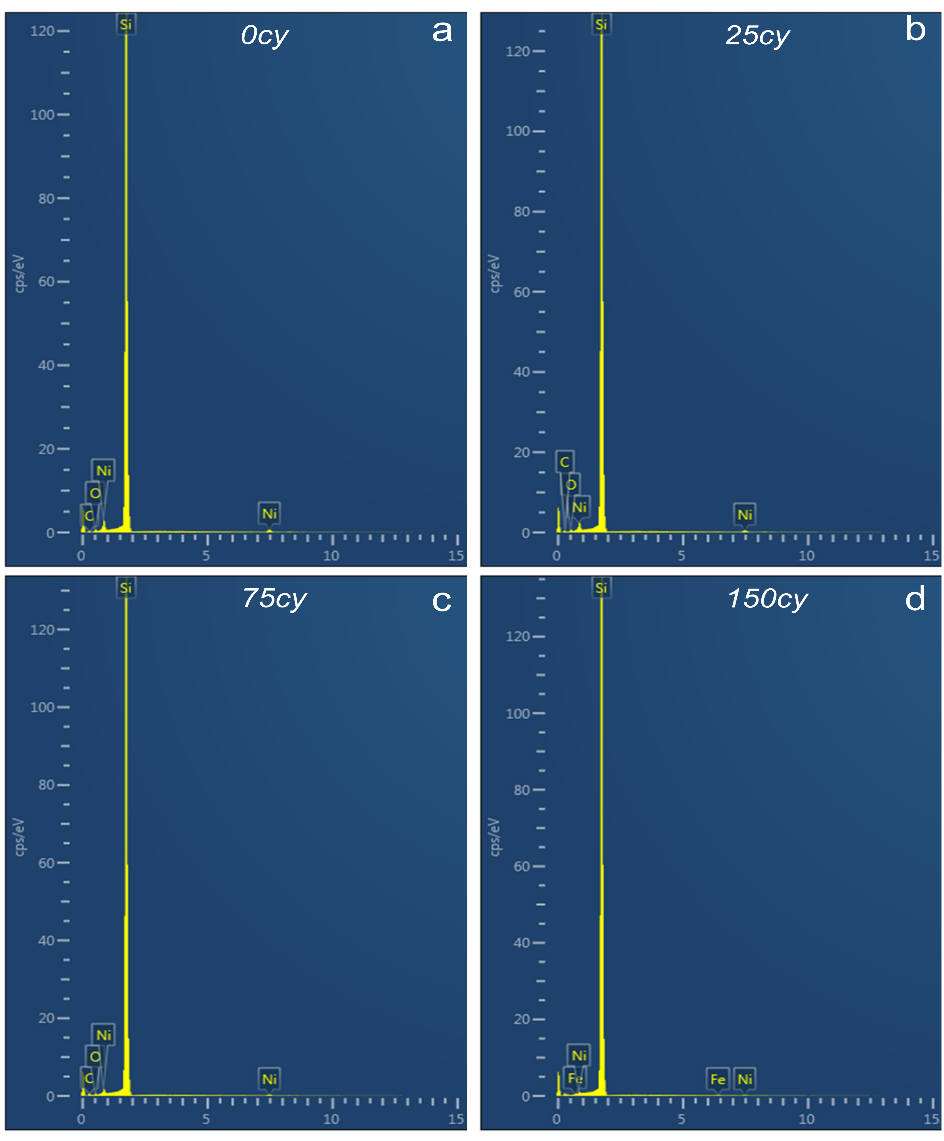


**Supplementary Figure 8. EDS spectra**. a) *p^+^-*Si/SiO_x_/Ni/NiFePB-*0cy*, b) *p^+^-*Si/SiO_x_/Ni/NiFePB-*25cy*, c) *p^+^-*Si/SiO_x_/Ni/NiFePB-*75cy* and d) *p^+^-*Si/SiO_x_/Ni/NiFePB-*150cy*.


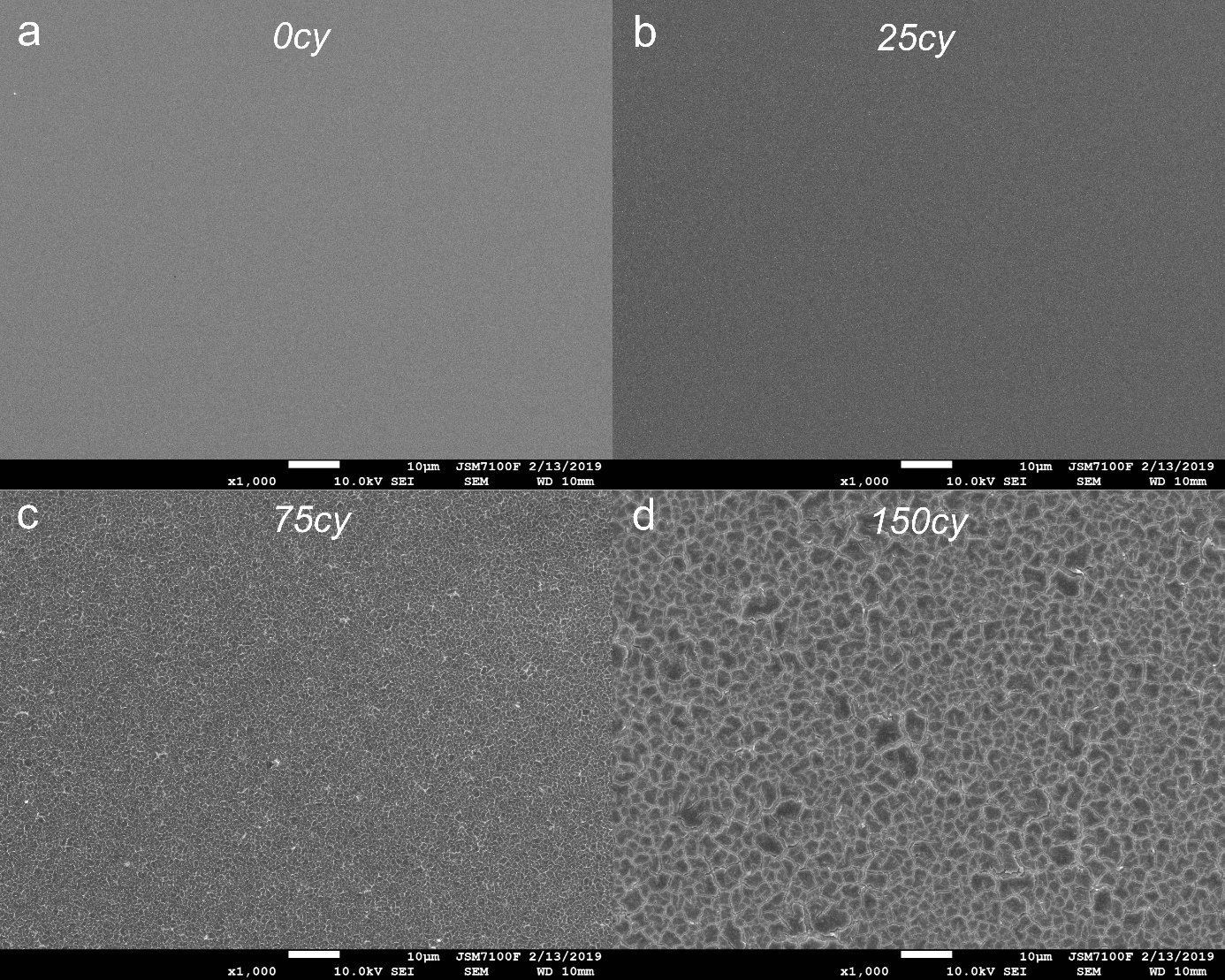


**Supplementary Figure 9. SEM top view images at a 1k magnification.** a) *p^+^-*Si/SiO_x_/Ni/NiFePB-*0cy*, b) *p^+^-*Si/SiO_x_/Ni/NiFePB-*25cy*, c) *p^+^-*Si/SiO_x_/Ni/NiFePB-*75cy* and d) *p^+^-*Si/SiO_x_/Ni/NiFePB-*150cy*.


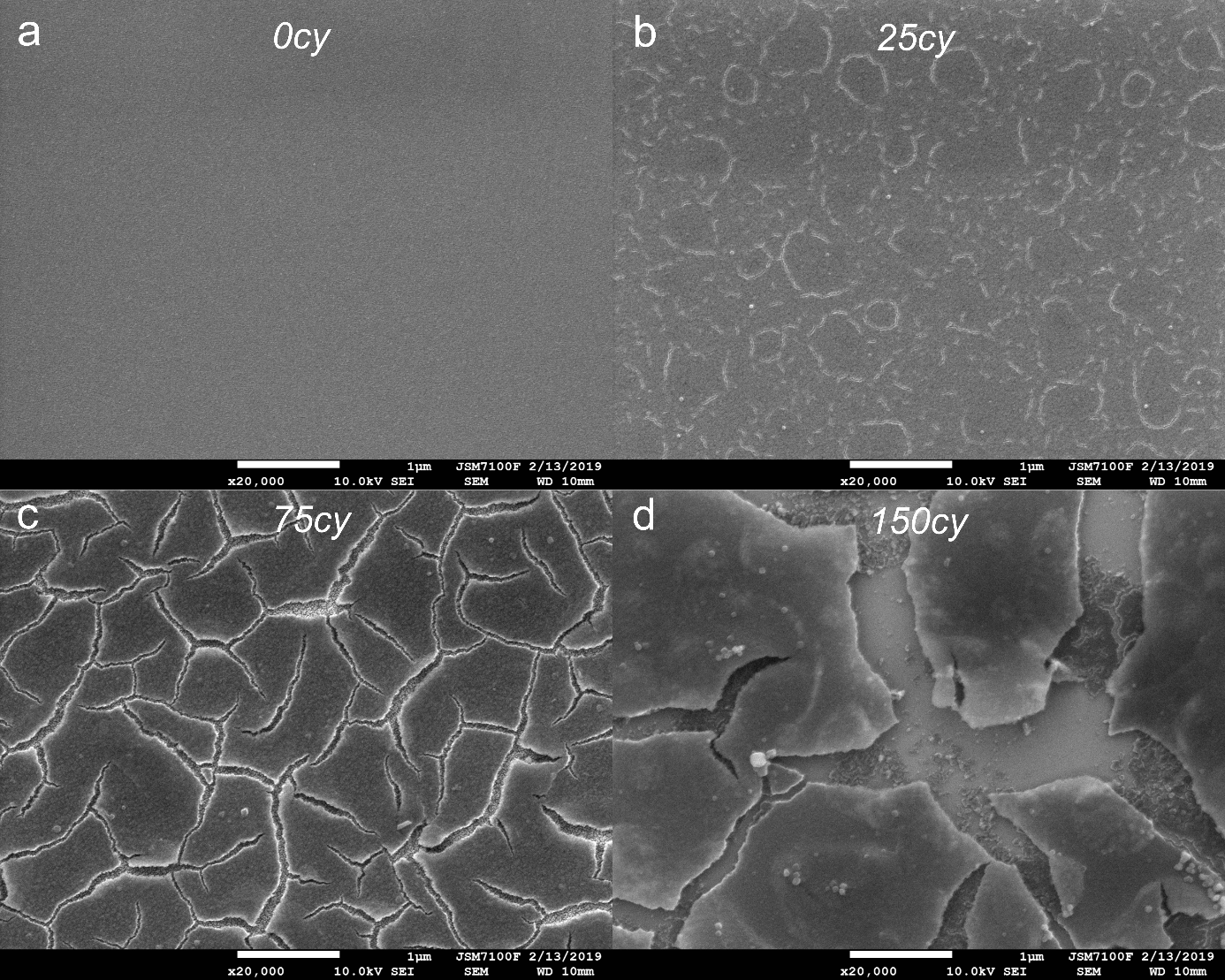


**Supplementary Figure 10. SEM top view images at a 20k magnification.** a) *p^+^-*Si/SiO_x_/Ni/NiFePB-*0cy,* b) *p^+^-*Si/SiO_x_/Ni/NiFePB-*25cy*, c) *p^+^-*Si/SiO_x_/Ni/NiFePB-*75cy* and d) *p^+^-*Si/SiO_x_/Ni/NiFePB-*150cy*.


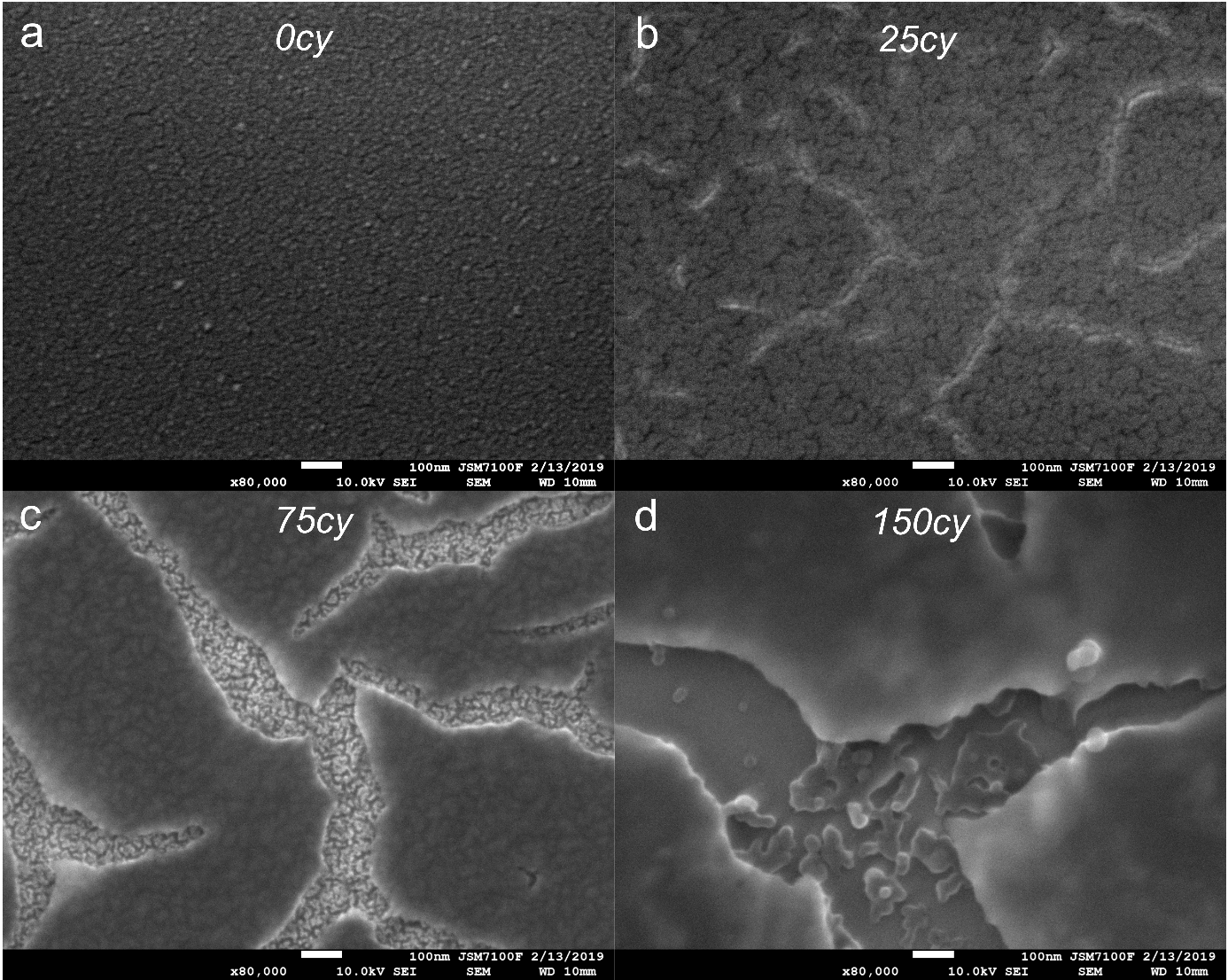


**Supplementary Figure 11. SEM top view images at a 80k magnification.** a) *p^+^-*Si/SiO_x_/Ni/NiFePB-*0cy*, b) *p^+^-*Si/SiO_x_/Ni/NiFePB*-25cy*, c) *p^+^-*Si/SiO_x_/Ni/NiFePB*-75cy* and d) *p^+^-*Si/SiO_x_/Ni/NiFePB-*150cy*.


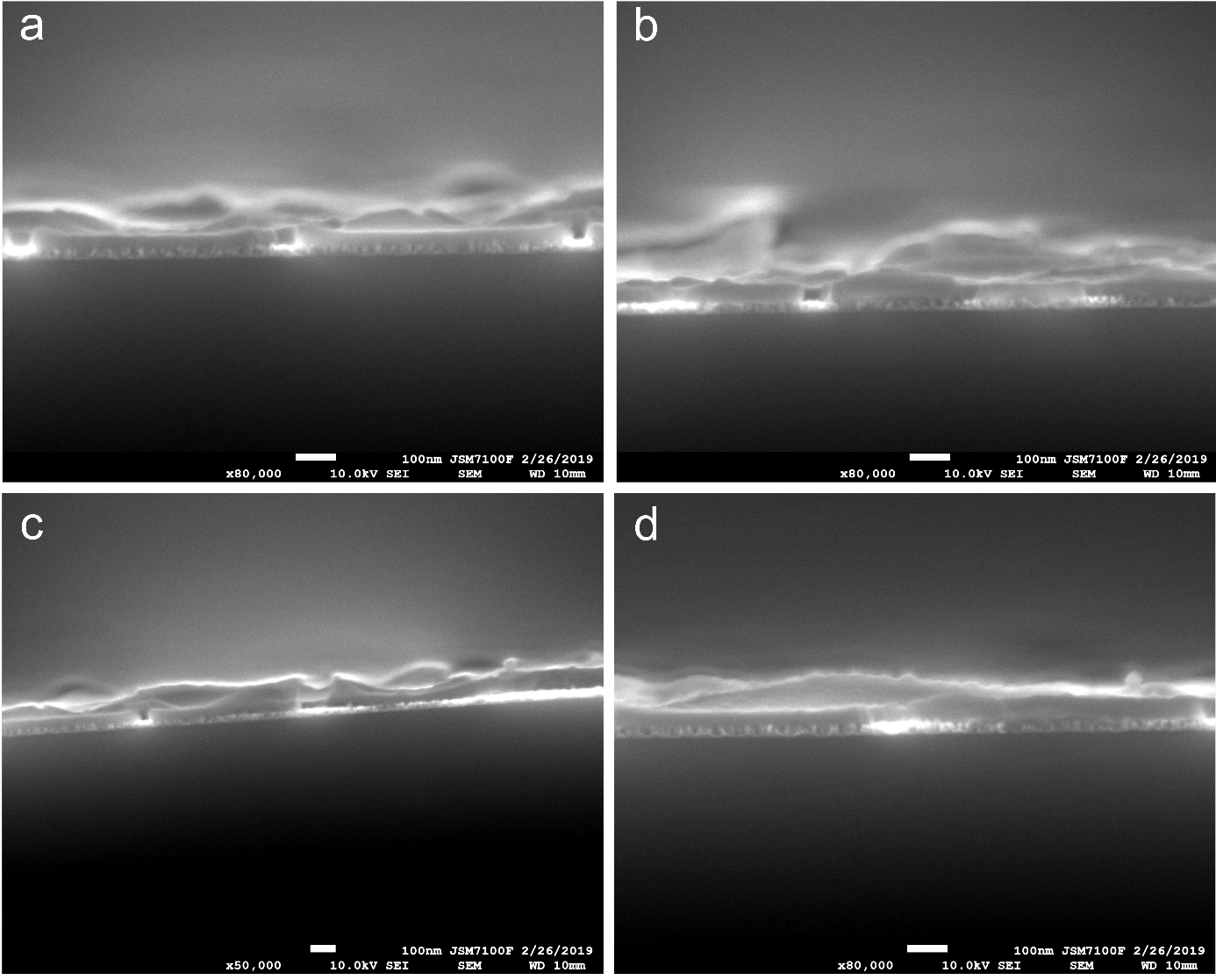


**Supplementary Figure 12. SEM cross-sections.** Images of *p^+^-*Si/SiO_x_/Ni/NiFePB-*75cy*.

**
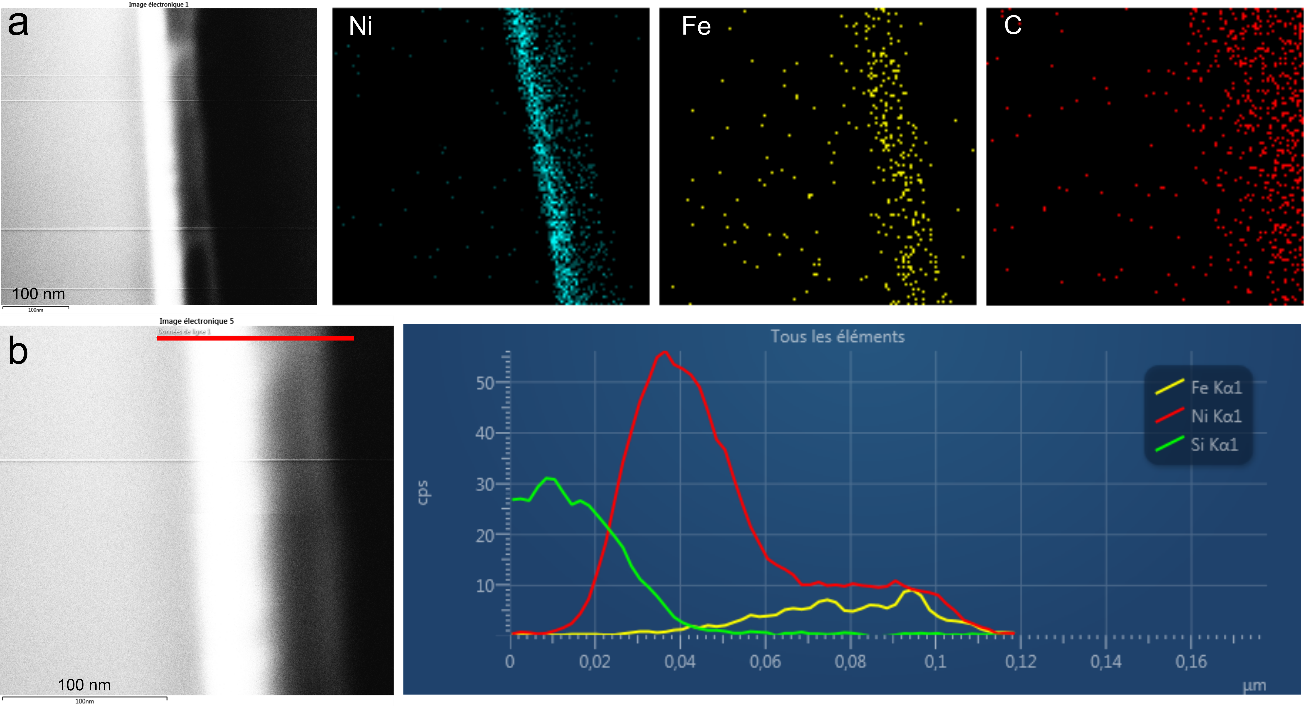
**

**Supplementary Figure 13. Elemental analysis over a cross-section.** STEM images (left) and EDS analysis of *p^+^-*Si/SiO_x_/Ni/NiFePB-*75cy*, a) Ni, Fe and C mapping and b) profiles for Ni, Fe and Si peak intensities along the red line in the STEM image.


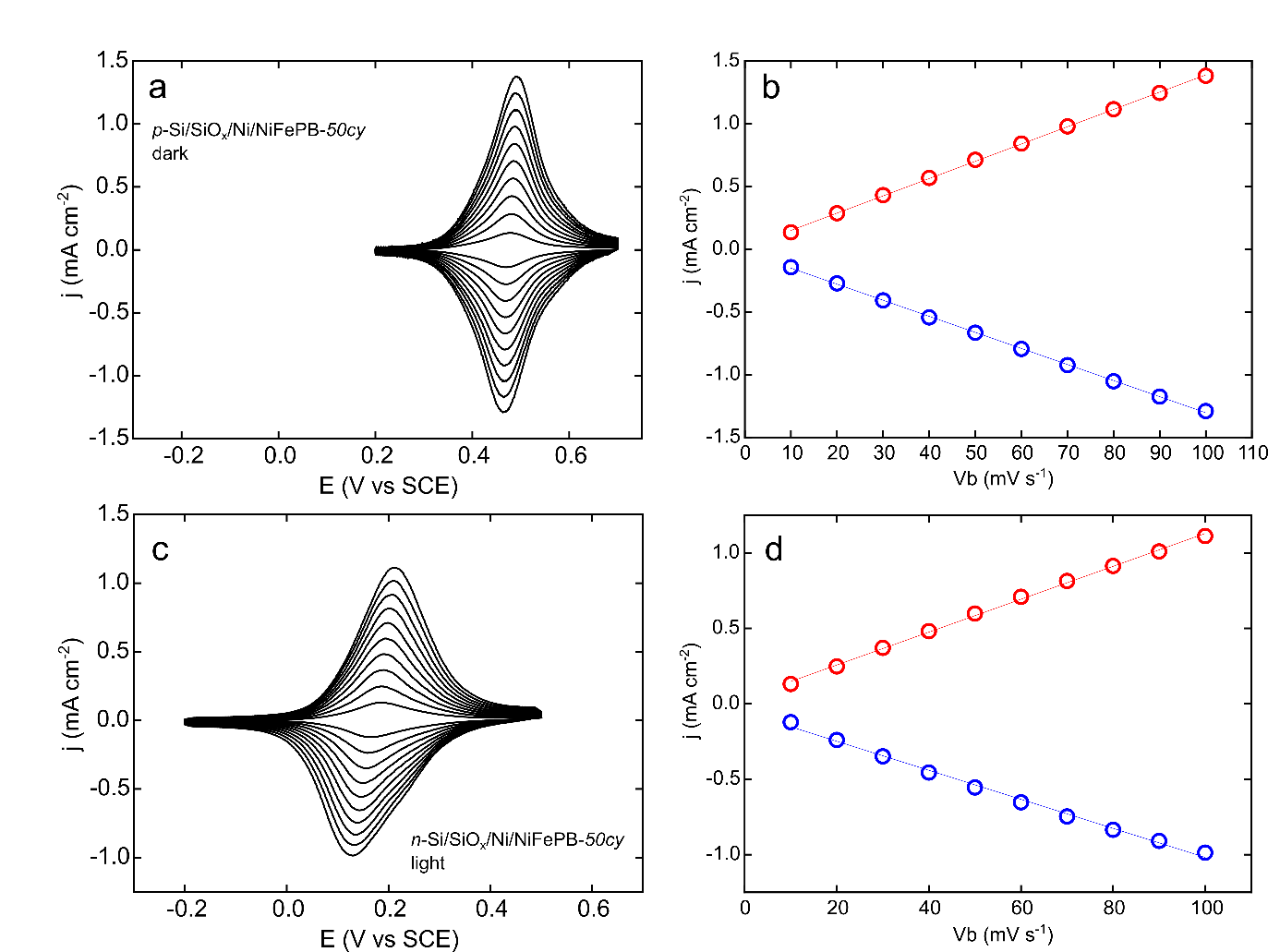


**Supplementary Figure 14. Effect of the scan rate on the CVs.** a) CVs recorded for *p^+^-*Si/SiO_x_/Ni/NiFePB-*50cy* in Ar-degassed 1 M KCl in the dark at scan rates varying from 10 to 100 mV s^-1^. b) Corresponding plots of the anodic (red) and the cathodic (blue) peak currents as a function of the scan rate (lines are linear fits). c) CVs recorded for *n*-Si/SiO_x_/Ni/NiFePB-*50cy* in Ar-degassed 1 M KCl under illumination at scan rates varying from 10 to 100 mV s^-1^. d) Corresponding plots of the anodic (red) and the cathodic (blue) peak current as a function of the scan rate (lines are linear fits).


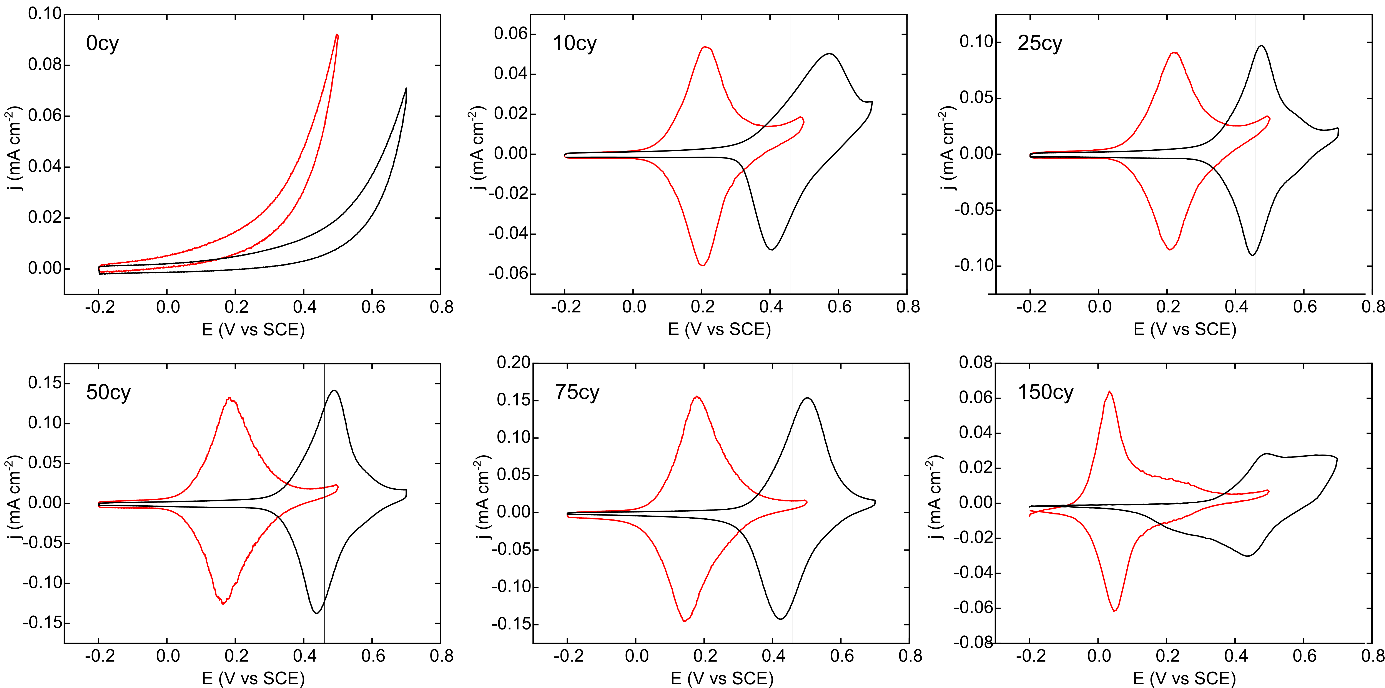


**Supplementary Figure 15. Dark and light CVs recorded on *n*-Si-based MIS electrodes.** CVs recorded in the dark (black curves) and under illumination (red curves) on *n*-Si/SiO_x_/Ni/NiFePB-*0cy*, *n*-Si/SiO_x_/Ni/NiFePB-*10cy*, *n*-Si/SiO_x_/Ni/NiFePB-*25cy*, *n*-Si/SiO_x_/Ni/NiFePB-*50cy*, *n*-Si/SiO_x_/Ni/NiFePB-*75cy and n*-Si/SiO_x_/Ni/NiFePB-*150cy*. The value *E*°’ = 0.46 V vs SCE is represented by a black line.


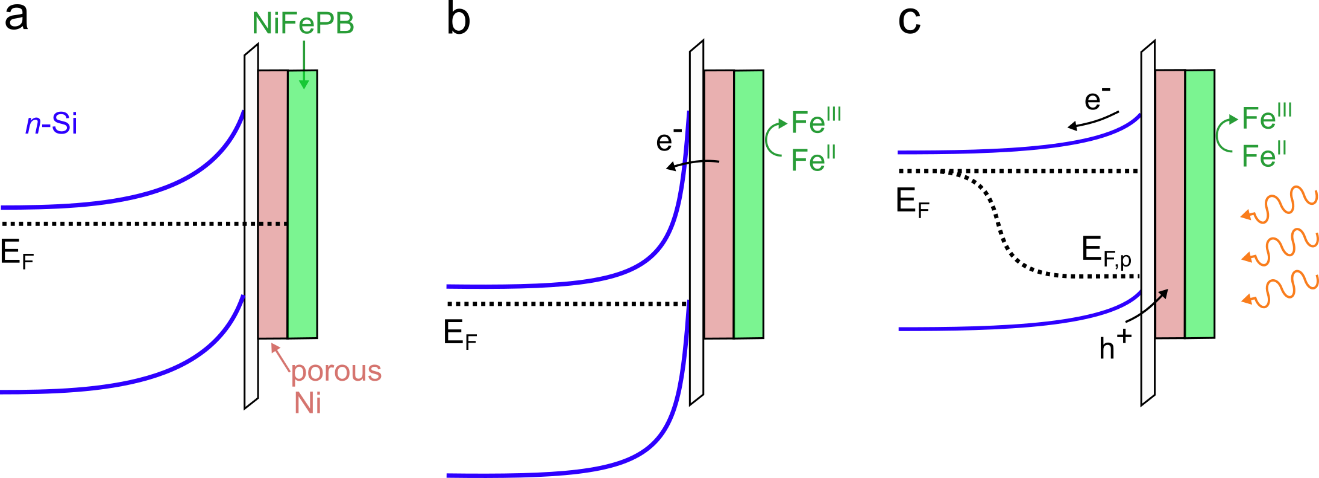


**Supplementary Figure 16. Schematic band diagrams.** a) *n*-Si/SiO_x_/Ni/NiFePB surface at equilibrium in the dark. b) *n*-Si/SiO_x_/Ni/NiFePB surface in the dark with an important applied anodic bias. c) *n*-Si/SiO_x_/Ni/NiFePB in operation under illumination.


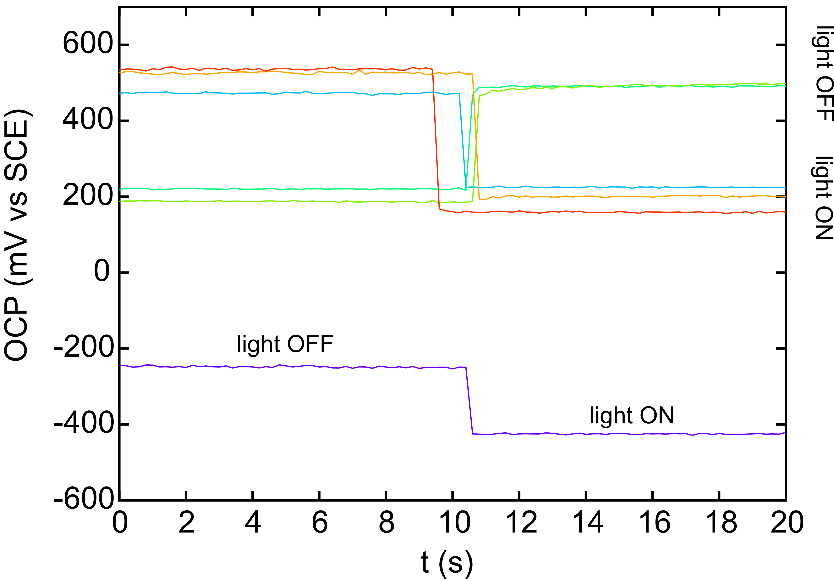


**Supplementary Figure 17. Dark and light OCP.** These measurements were recorded in 1 M KCl for *n*-Si/SiO_x_/Ni/NiFePB, prepared with different number of cycles ((purple) *0cy*, (light blue) *25cy*, (cyan) *50cy*, (light green) *75cy*, (yellow) *100cy,* (red) *150cy*).


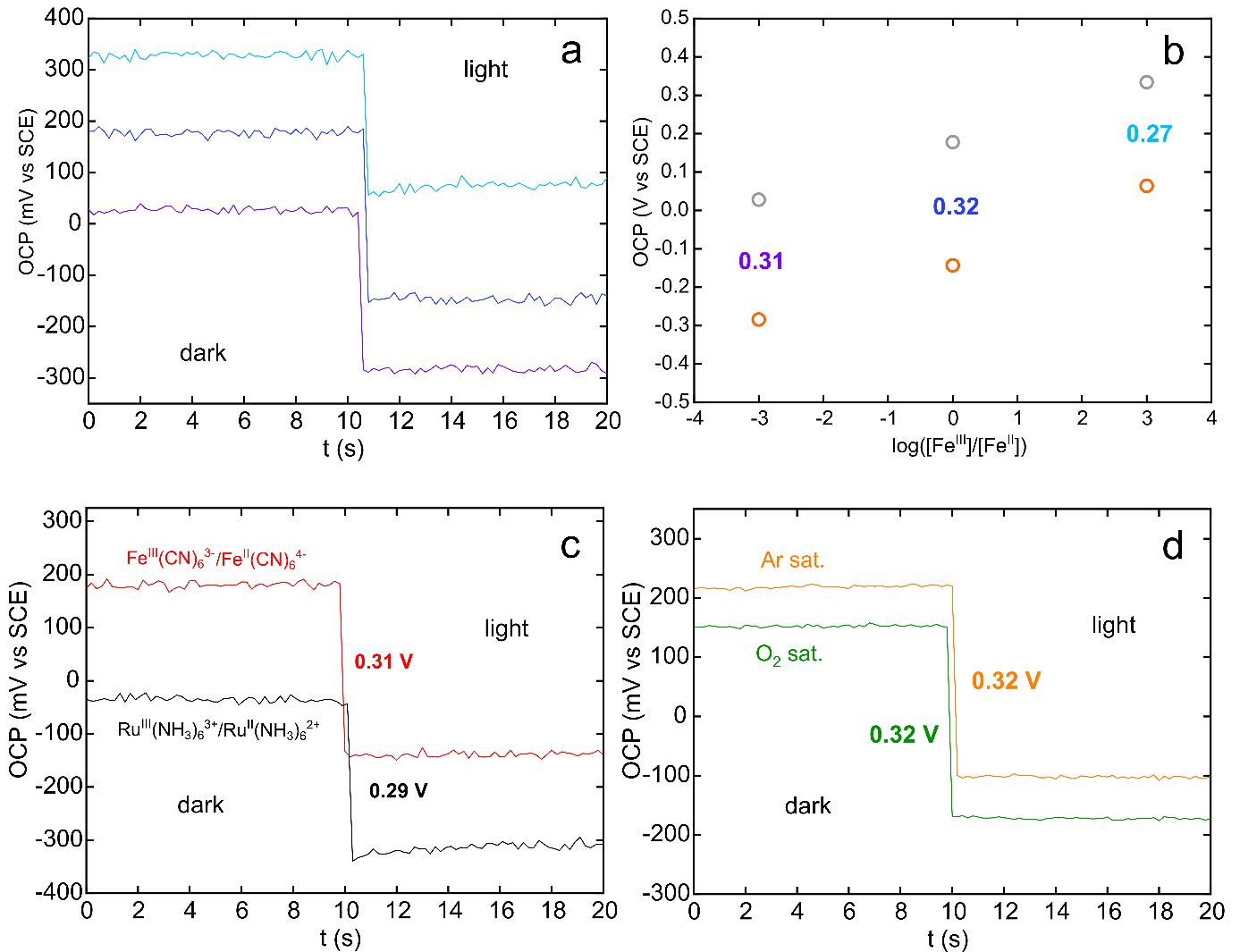


**Supplementary Figure 18. Dark and light OCP measurements recorded on *n*-Si/SiO_x_/Ni/NiFePB*-75cy*.** a) In a Ar-degassed 0.1 M KCl solution containing K_3_Fe(CN)_6_/K_4_Fe(CN)_6_ at the following concentrations: (light blue) 0.1 M/0.1 mM; (dark blue) 0.5 mM/0.5 mM and (purple) 0.1 mM / 0.1 M. b) Corresponding plots of the OCPs as a function of the ratio of the relative concentration of K_3_Fe(CN)_6_ and K_4_Fe(CN)_6_. c) Dark and light OCP measurements recorded on *n*-Si/SiO_x_/Ni/NiFePB*-75cy*, in a Ar-degassed 0.1 M KCl solution containing (red) 0.5 mM/0.5 mM K_3_Fe(CN)_6_/K_4_Fe(CN)_6_ and (black) 0.5 mM/0.5 mM Ru(NH_3_)_6_Cl_3_/ Ru(NH_3_)_6_Cl_2_. d) Dark and light OCP measurements recorded on *n*-Si/SiO_x_/Ni/NiFePB*-75cy*, in (yellow) Ar-degassed 0.1 M KCl solution and in a (light green) O_2_-degassed 1 M KCl. The values of photovoltages are displayed in bold.


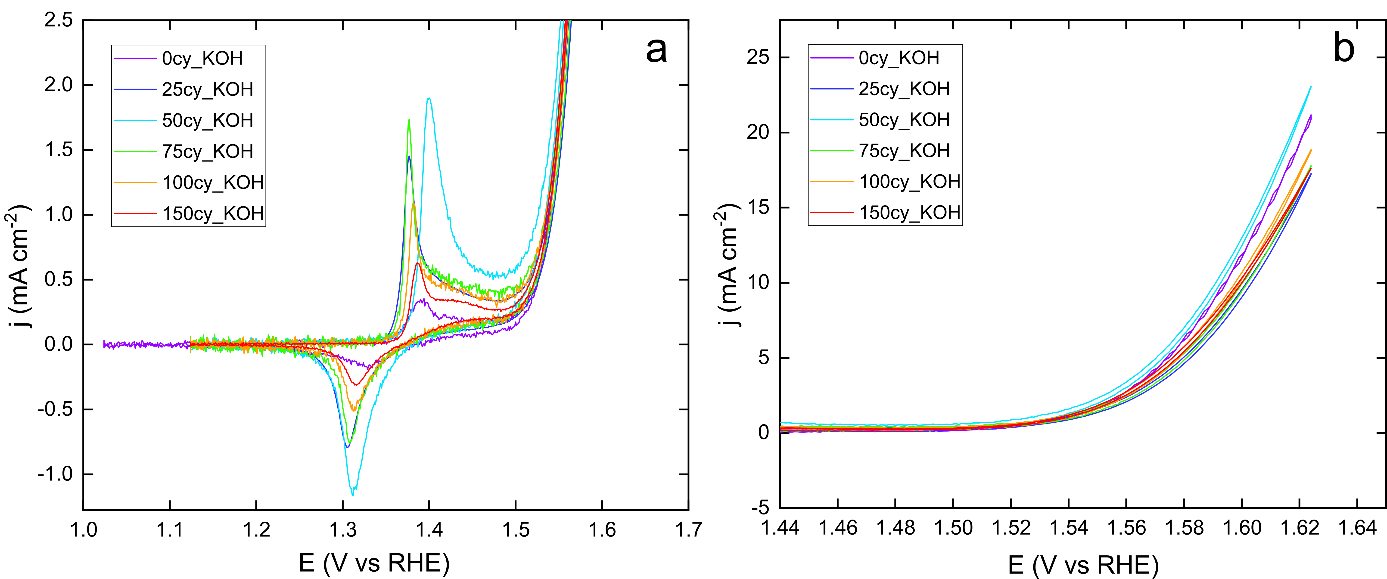


**Supplementary Figure 19**. **CVs recorded in 1 M KOH.** These measurements were performed at 10 mV s^-1^ on *p^+^-*Si/SiO_x_/Ni/NiFePB MIS anodes (the color code is displayed in the inset) these curves show: a) the quasi-reversible electrochemical waves corresponding to the Ni^III^/Ni^II^ redox system and b) the OER reaction.


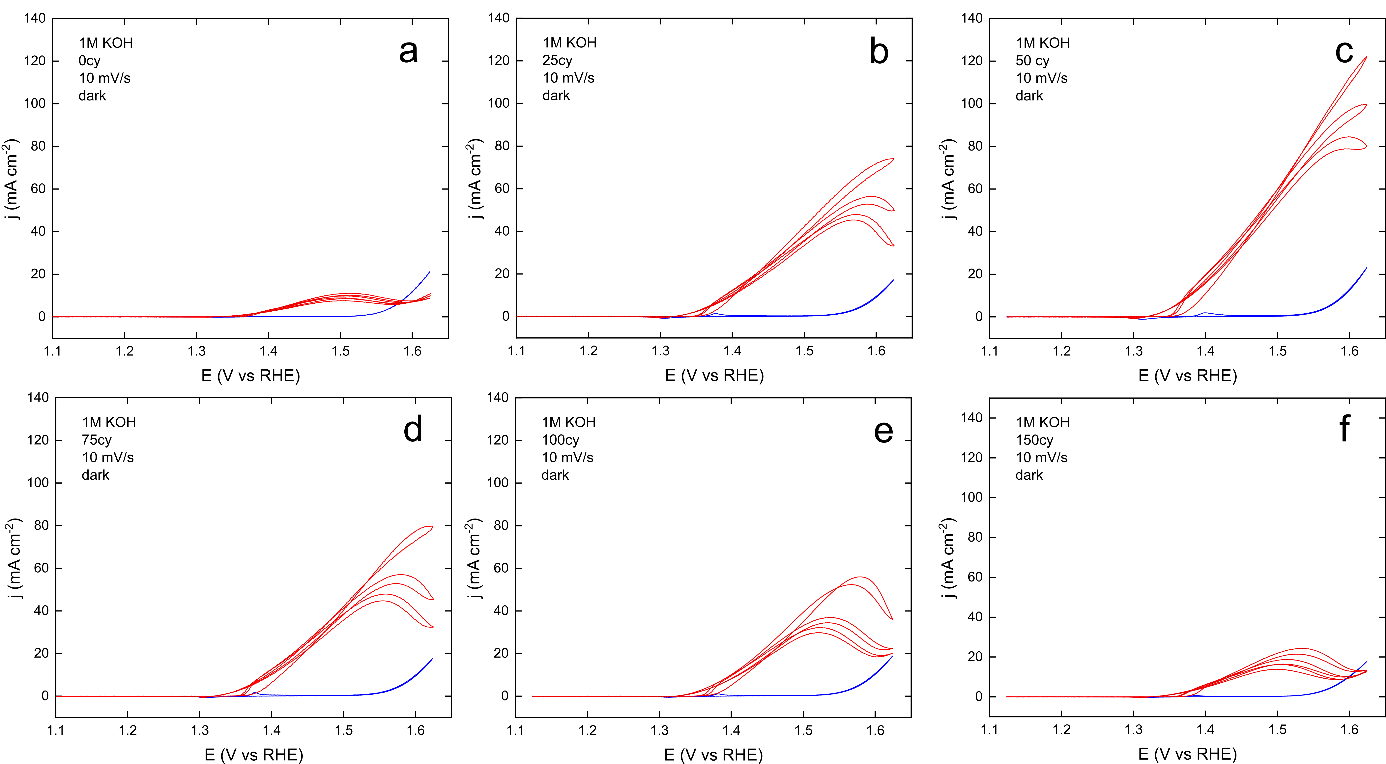


**Supplementary Figure 20.** **Influence of the urea on non-photoactive junctions.** CVs recorded in 1 M KOH in the absence of urea (blue CVs) and in the presence of 0.33 M urea (red CVs, three first scans) at 10 mV s^-1^ on *p^+^-*Si/SiO_x_/Ni/NiFePB MIS anodes. The CV were recorded on: a) *p^+^-*Si/SiO_x_/Ni*-0cy*, b) *p^+^-*Si/SiO_x_/Ni/NiFePB*-25cy*, c) *p^+^-*Si/SiO_x_/Ni/NiFePB*-50cy*, d) *p^+^-*Si/SiO_x_/Ni/NiFePB*-75cy*, e) *p^+^-*Si/SiO_x_/Ni/NiFePB*-100cy* and f) *p^+^-*Si/SiO_x_/Ni/NiFePB*-150cy*.


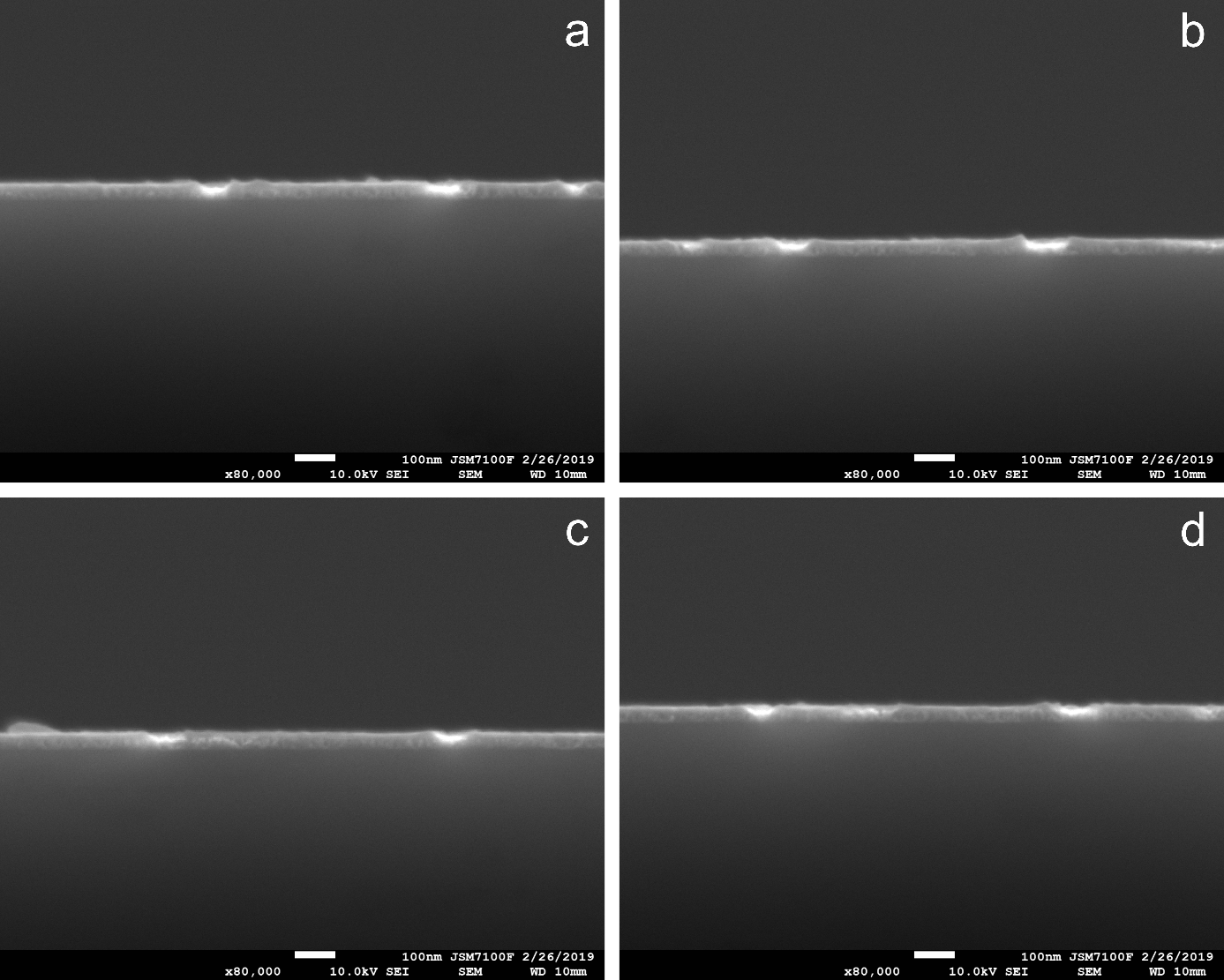


**Supplementary Figure 21. Cross-sections after UOR.** SEM cross-section images at a 80k magnification of *p^+^-*Si/SiO_x_/Ni/NiFePB-*75cy* after UOR.


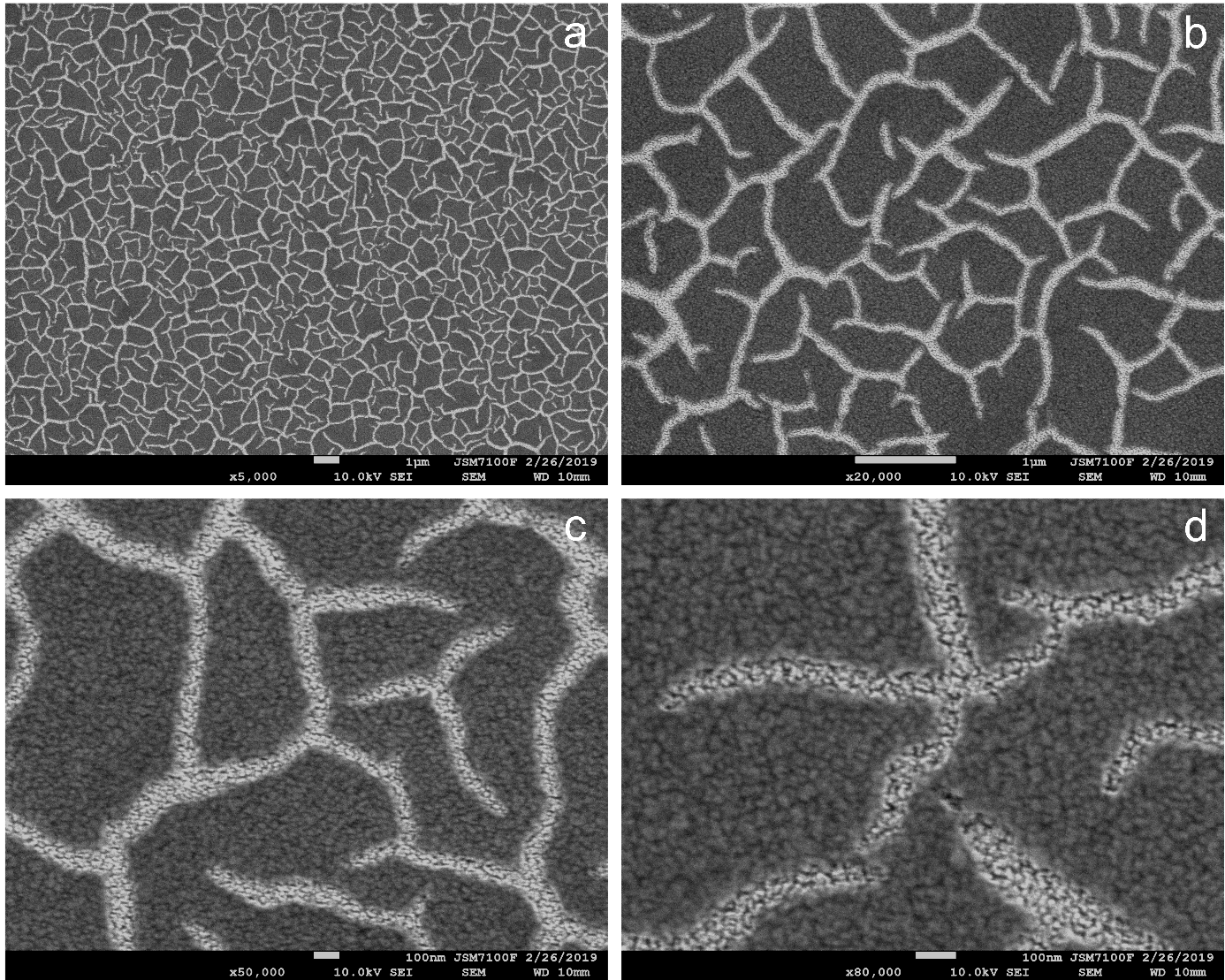


**Supplementary Figure 22. Top views after UOR.** SEM top view images of *p^+^-*Si/SiO_x_/Ni/NiFePB-*75cy* after UOR.


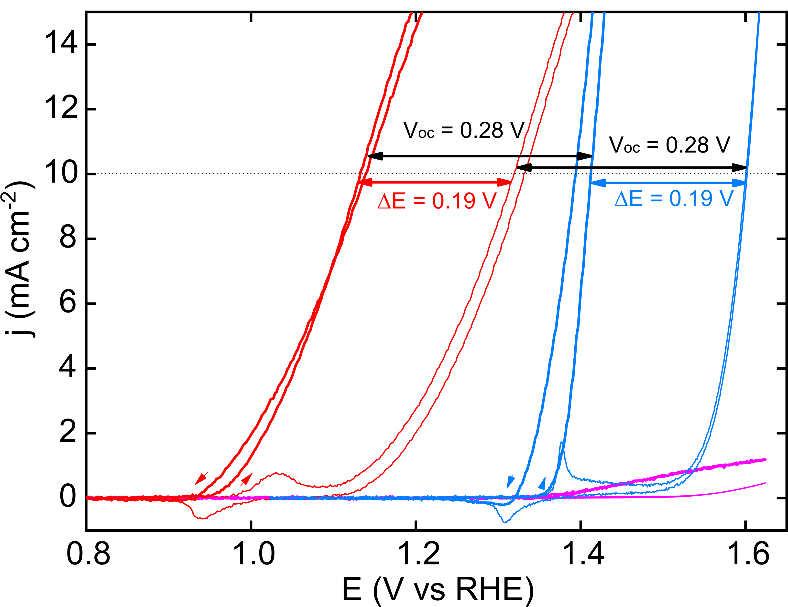


**Supplementary Figure 23.** **Cyclic voltammetry at different Si/SiO_x_/Ni/Ni(OH)_2_-*75cy* surfaces.** CVs of (light blue) *p^+^-*Si/SiO_x_/Ni/Ni(OH)_2_-*75cy* in the dark, (pink) *n*-Si/SiO_x_/Ni/Ni(OH)_2_-*75cy* in the dark and (red) *n*-Si/SiO_x_/Ni/Ni(OH)_2_-*75cy* under simulated sunlight; the CVs recorded in 1 M KOH are represented by thin lines and the CVs recorded in 1 M KOH + 0.33 M urea are represented by thick lines. The values of *V*_oc_ measured at 10 mA cm^-2^ are represented in black, the values of Δ*E* are represented in red for *n*-Si/SiO_x_/Ni/Ni(OH)_2_-*75cy* under illumination and blue for *p^+^-*Si/SiO_x_/Ni/Ni(OH)_2_-*75cy* in the dark.


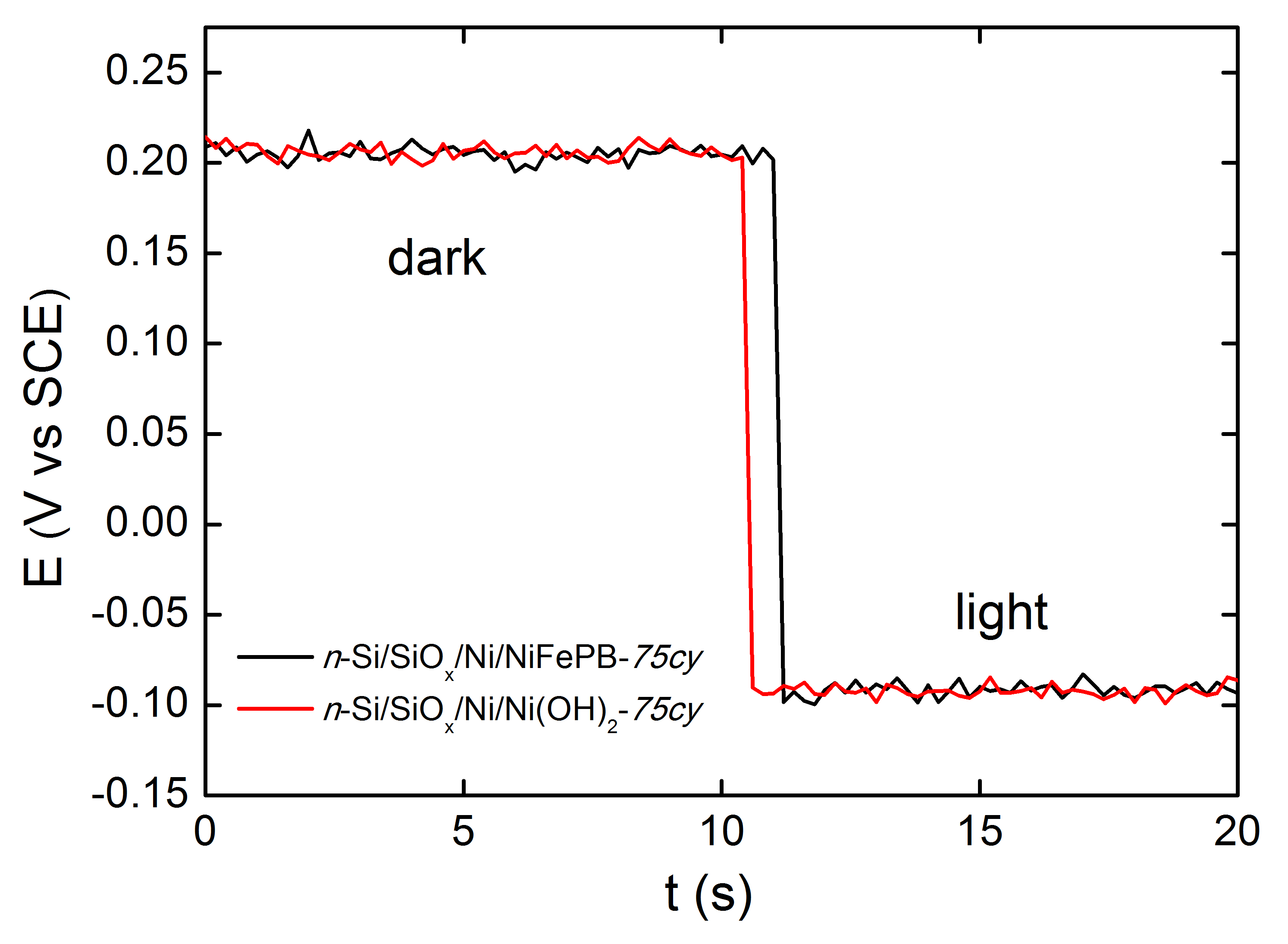


**Supplementary Figure 24. Effect of UOR on OCP.** Dark and light (simulated sunlight) OCP measurements recorded in a Ar-degassed 0.1 M KCl solution containing K_3_Fe(CN)_6_/K_4_Fe(CN)_6_ (0.5 mM/0.5 mM) on *n*-Si/SiO_x_/Ni/NiFePB-*75cy* (black curve) and *n*-Si/SiO_x_/Ni/Ni(OH)_2_-*75cy* (red curve).


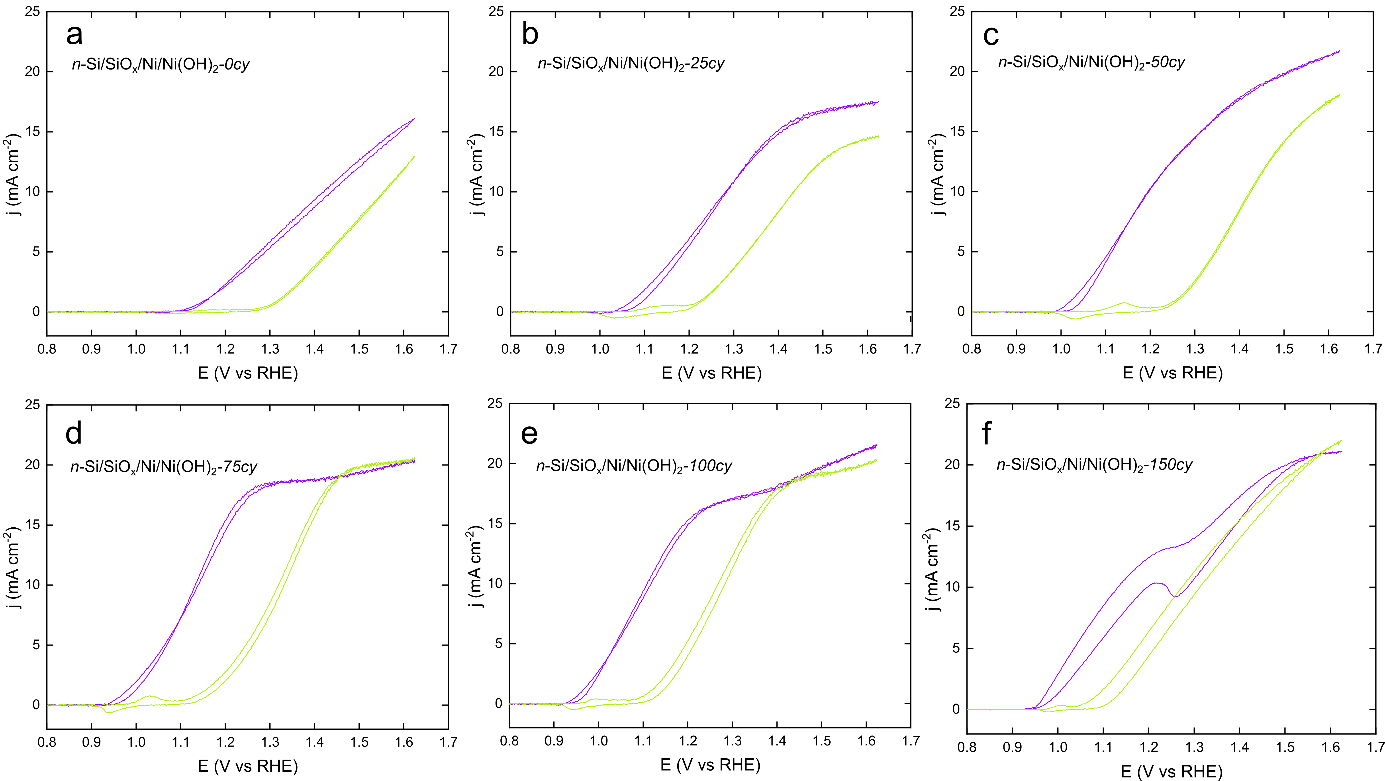


**Supplementary Figure 25.** **Influence of the urea on photoactive junctions.** a) CVs of *n*-Si/SiO_x_/Ni/Ni(OH)_2_, prepared with a) *0cy*, b) *25cy*, c) *50cy*, d) *75cy*, e) *100cy* and f) *150cy* in 1 M KOH at 10 mV s^-1^ under illumination (light green) in the absence of urea and (purple) in the presence of 0.33 M urea.


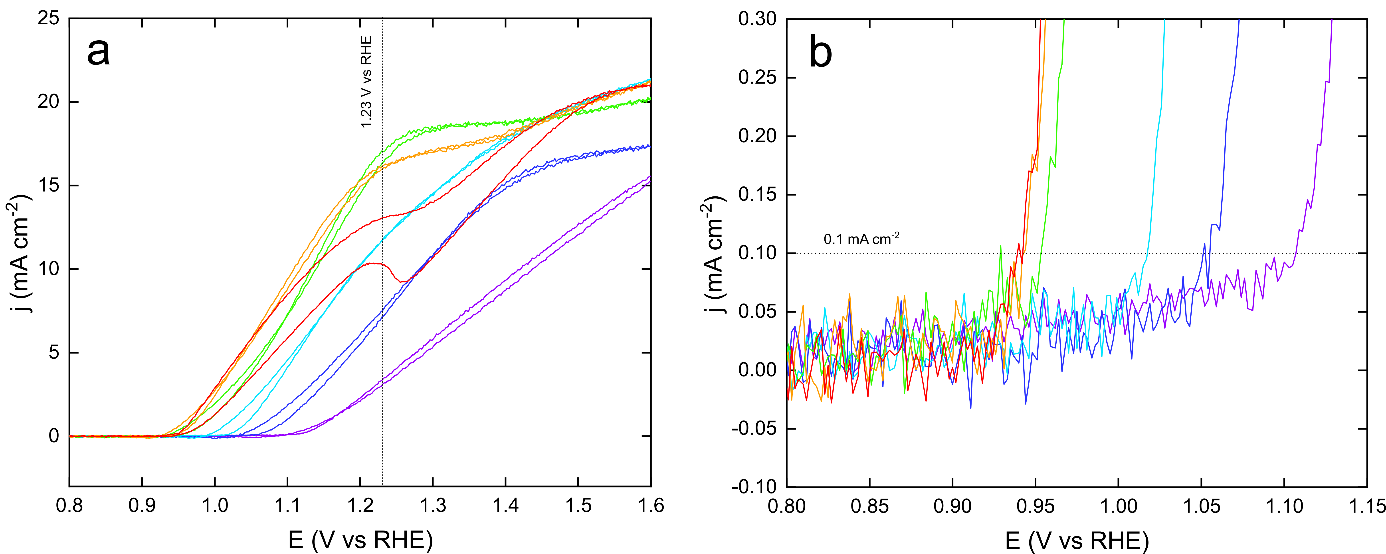


**Supplementary Figure 26. Influence of the cycle number.** a) CVs and b) LSVs of *n*-Si/SiO_x_/Ni/Ni(OH)_2_, prepared with different number of cycles ((purple) *0cy*, (light blue) *25cy*, (cyan) *50cy*, (light green) *75cy*, (yellow) *100cy,* (red) *150cy*) under simulated sunlight in 1 M KOH/0.33 M KOH at 10 mV s^-1^.


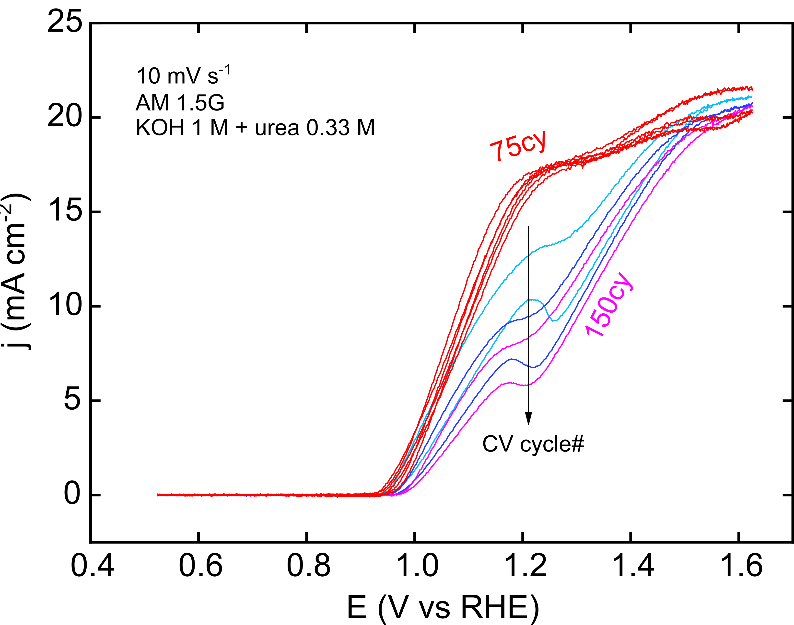


**Supplementary Figure 27. Investigation of the stability by voltammetry.** Three consecutive CV cycles recorded at 10 mV s^-1^ in 1 M KOH + 0.33 M urea on (red) *n*-Si/SiO_x_/Ni/Ni(OH)_2_-*75cy* and *n*-Si/SiO_x_/Ni/Ni(OH)_2_-*150cy*.


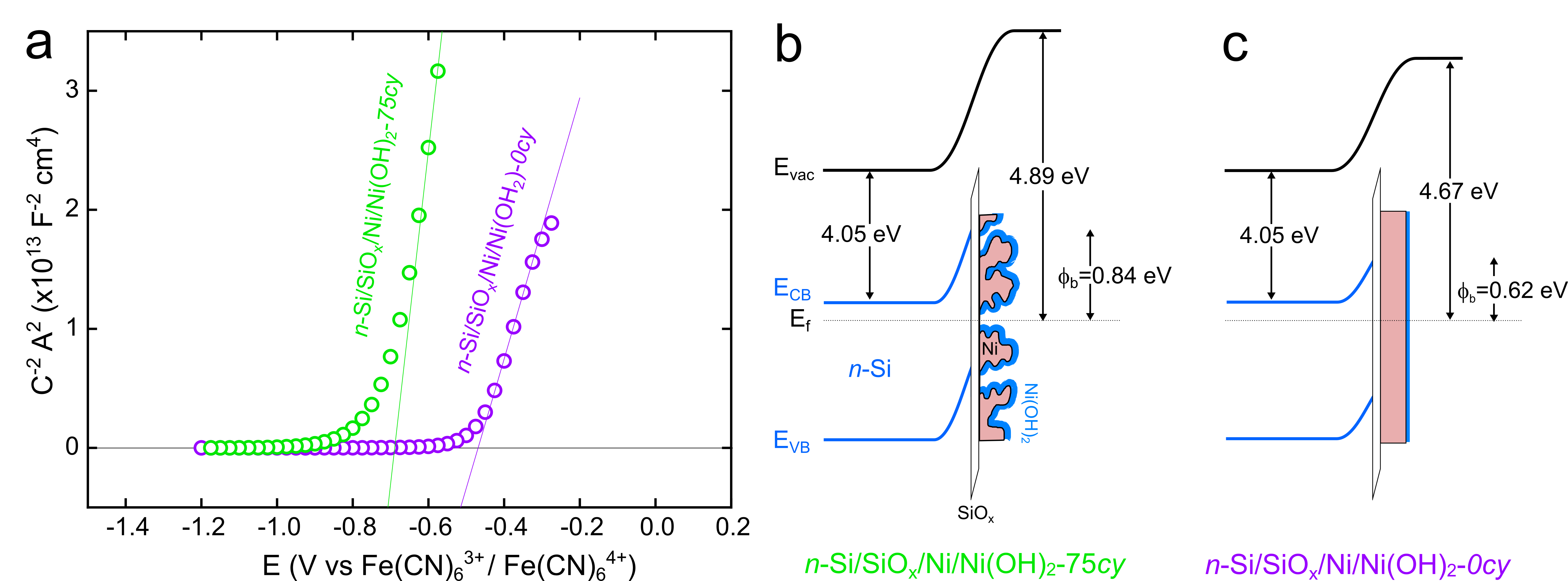


**Supplementary Figure 28. Determination of the band diagrams.** a) M-S plots of (light green) *n*-Si/SiO_x_/Ni/Ni(OH)_2_-*75cy* and (purple) *n*-Si/SiO_x_/Ni/Ni(OH)_2_-*0cy*. b) Band diagram for *n*-Si/SiO_x_/Ni/Ni(OH)_2_-*75cy*. c) Band diagram for *n*-Si/SiO_x_/Ni/Ni(OH)_2_-*0cy*.


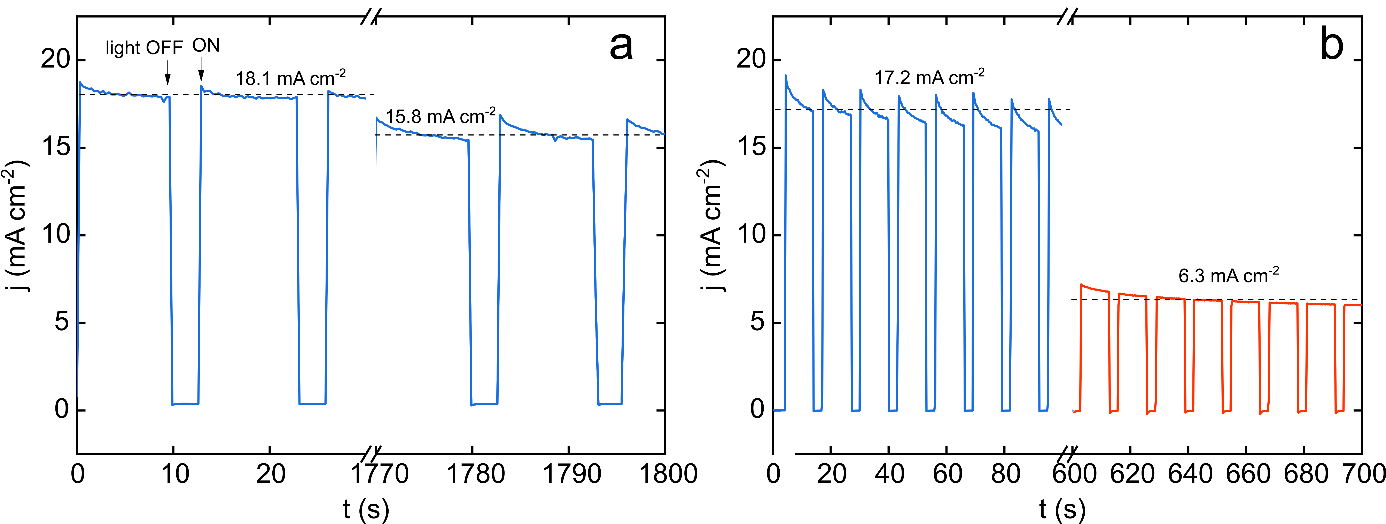


**Supplementary Figure 29**. **Electrolyses at imposed potential.** Chronoamperometry curves for UOR on *n*-Si/SiO_x_/Ni/Ni(OH)_2_-*75cy* recorded at 1.40 V (a) and 1.23 V (b) under intermittent illumination in 0.33 M urea/1 M KOH. In (b), the electrolyte was replaced at 600 s by 1 M KOH.

# Supplementary Tables

**Supplementary** **Table 1.** Values of apparent standard potential *E*^0’^ calculated from the average of the anodic peak *E*_pa_ and the cathodic peak *E*_pc_ potentials of the CVs recorded in the dark for Si/SiO_x_/Ni/NiFePB electrodes in 1 M KCl at a scan rate of 10 mV s^-1^ as a function of Si type and the cycle number (*#cy*).

| **Si type** | ***#cy*** | **ID** | ***E*_pa_ (mV vs SCE)** | ***E*_pc_  (mV vs SCE)** | **Δ*E*_p_  (mV vs SCE)** | ***E*^0'^ (mV vs SCE)** | **Average  (mV vs SCE)** |
| --- | --- | --- | --- | --- | --- | --- | --- |
| **p** | 25 | p5_2801 | 477 | 471 | 7 | 474 | 471 |
|  |  | p6_2501 | 475 | 462 | 13 | 468 |  |
|  | 50 | p4_2801 | 476 | 469 | 7 | 473 |  |
|  |  | P2_0205 | 478 | 469 | 9 | 474 |  |
|  | 75 | p3_2801 | 481 | 463 | 18 | 472 |  |
|  | 100 | p1_2801 | 491 | 453 | 38 | 472 |  |
|  |  | p1_0206 | 476 | 454 | 22 | 465 |  |
|  | 150 | p1_2801 | 475 | 467 | 9 | 471 |  |
| **n** | 25 | n5_2805 | 478 | 449 | 29 | 463 | 463 |
|  | 50 | n4_2805 | 488 | 438 | 50 | 463 |  |
|  | 75 | n6_2802 | 502 | 422 | 80 | 462 |  |
|  | 100 | n2_2805 | 485 | 445 | 40 | 465 |  |
|  | 150 | n2_2501 | 489 | 438 | 51 | 464 |  |

**Supplementary** **Table 2.** List of the catalysts, electrolytes and approximate onset potentials employed for UOR in the literature (Edep = electrodeposited)

| **Reference** | **Catalyst** | **Electrolyte** | **Onset (V vs RHE)** |
| --- | --- | --- | --- |
| Botte *et al.* Chem. Commun. 2009, 4859 | Edep Ni^0^ | 5 M KOH  0.33 M urea | 1.3 |
| Botte *et al.* Electrochim. Acta 2012, 292 | Edep Ni^0^ | 5 M KOH 0.33 M urea | 1.3 |
| Botte *et al.* J. Power Sources 2011, 9579 | Pt, Pt-Ir, Rh, Ru Edep on Ni plate | 1 M KOH 0.33 M urea | 1.4 |
| Botte *et al.* Electrochem. Commun. 2011, 1135 | Ni(OH)_2_ exfoliated by a wet process | 5 M KOH 0.33 M urea | 1.3 |
| Botte *et al.* Electrochim. Acta 2014, 266 | Ni nanowires Edep in a porous template | 1M KOH 0.33 urea | >1.37 |
| Schechter *et al.* ChemElectroChem 2017, 1037 | Ni Edep on Sn dendrites | 1 M KOH  0.33 M urea | 1.4 |
| Botte *et al.* Electrochim. Acta 2013, 660 | Ni(OH)_2_ Edep on gold | 5 M KOH 1 M urea | 1.3 |
| Botte *et al.* ECS Electrochem. Lett. 2014, H29 | Ni(OH)_2_/ carbone paper | 5 M KOH 1M urea | 1.36 |
| Tao *et al.* New J. Chem. 2017, 4190 | Hydrothermal NiFe(OH)_2_ double hydroxydes | 1 M NaOH 0.33 M urea | 1.43 |
| Zheng et al. Electrochim. Acta 2014, 194 | Ni(OH)_2_ Edep on PS beads | 1M KOH 0.33 urea | 1.4 |
| Wu *et al.* Electrochem. Commun. 2013, 21 | Ni(OH)_2_ Edep on ZnO nanorods on Ni foam | 1M KOH 0.33 urea | 1.37 |
| Botte *et al.* J. Power Source 2012, 498 | Ni(OH)_2_ nanoribbons on glassy carbon | 5 M KOH 0.33 M urea | 1.4 |
| Botte *et al.* Electrochim. Acta 2013, 732 | Ni-graphene prepared by dispersion of graphene oxide and cathodic deposition of Ni | 1 M KOH 0.33 M urea | 1.35 |
| Chen *et al.* ACS App. Mater. Interfaces 2018, 41338 | Ni-Co-WC-MCNT Wet chem | 1 M KOH 0.33 M urea | 1.27 |
| Valles *et al.* App. Surf. Sci. 2016, 816 | Edep CoNi films activated by CV in NaOH | 0.5 M NaOH  0.1 M urea | 1.32 |
| Botte *et al.* J. Appl. Electrochem. 2015, 1217 | NiCo bimetallic nanowires Edep in porous template | 1 M KOH 0.33 M urea | 1.3 |
| Wu *et al.* Sci. Rep. 2014, 5863 | NiCo bimetallic deposits on carbon | 1 M KOH 0.33 M urea | 1.2 |
| Wang *et al.* Nanoscale 2014, 1369 | NiCoO_2_ and Co_3_O_4_ spinels | 1 M KOH 0.33 M urea | 1.38 |
| Schechter *et al.* ACS App. Mater. Interfaces, 2016, 12176 | NiMnO_4_ spinels made by hydrothermal treatment deposited on Vulcan carbon | 1 M KOH 0.33 M urea | 1.32 |
| Botte *et al.* J. App. Electrochem. 2012, 925 | Rh Edep on Ni | 5 M KOH 0.33 M urea | 1.34 |
| Yang *et al.* J. Power Source 2014, 282 | Ni NP on WC on activated carbon | 1 M KOH 0.33 M urea | 1.32 |
| Schechter *et al.* ChemCatChem 2017, 3374 | NiCr | 1 M KOH 0.33 M urea | 1.34 |
| Stevenson *et al.* ACS Catal. 2016, 5044 | LaNiO_3_ perovskite | 5 M KOH 0.33 M urea | 1.3 |
| Liang *et al.* Electrochim. Acta 2015, 456 | NiMoO_4_ hydrothermal on Ni foam | 1 M KOH 0.33 M urea | 1.35 |
| Yu *et al.* Energy Environ. Sci. 2018, 1890 | NiMoO_4_ Nrods annealed (H_2_ or Ar) on Ni foam | 1 M KOH 0.5 M urea | 1.36 |

# Supplementary Notes

# Supplementary Note 1

In order to confirm the existence of the native oxide layer on the sputter-deposited Ni coating employed for the fabrication of the homogeneous MIS electrodes (*p^+^*-Si/SiO_x_/Ni-0cy), we performed an experiment where we milled the top part of the sputtered film by Ar inside the XPS chamber. After milling, the spectrum of Ni^0^ (green curve) was obtained, confirming that the oxide-rich Ni layer only exists on the top of the sputtered layer. To clearly illustrate that, the spectrum of the Ni 2p region of the Ar-milled surface is compared with that of the original, non-milled *p^+^*-Si/SiO_x_/Ni-0cy in Supplementary Figure 4. Note that the binding energy values measured for the three first peaks of the *p^+^*-Si/SiO_x_/Ni-0cy spectrum are in good agreement with the expected values of the Ni 2p_3/2_ peaks for Ni^0^, NiO and Ni(OH)_2_. In addition, the energy difference between the measured 2p_1/2_ and the 2p_3/2_ main peaks are in good agreement with those expected for Ni^0^ (17.4 eV) and Ni^II^ (17.8 eV).
