# Supplementary material for: Tailoring the photoelectrochemistry of catalytic metal-insulator-semiconductor (MIS) photoanodes by a dissolution method
Source: Nat Commun. 2019 Aug 6;10:3522. doi: 10.1038/s41467-019-11432-1 (PMC6684633; doi:10.1038/s41467-019-11432-1)
Supplement: Supplementary file 1 — Peer Review File [file 41467_2019_11432_MOESM1_ESM.pdf]

Editorial Note: This manuscript has been previously reviewed at another journal that is not operating a transparent peer review scheme. This document only contains reviewer comments and rebuttal letters for versions considered at *Nature Communications*

REVIEWERS' COMMENTS:

Reviewer #1 (Remarks to the Author):

The authors did a nice job responding to comments from both reviewers. The manuscript is now appropriate for publication in Nature Communications.
